# Degradation-Aware Multi-Stage Fusion for Underwater Image Enhancement

**DOI:** 10.3390/jimaging12010037

**Published:** 2026-01-08

**Authors:** Lian Xie, Hao Chen, Jin Shu

**Affiliations:** 1 School of Civil Engineering, Hunan City University, Yiyang 413000, China; xielian@whut.edu.cn; 2 School of Architecture and Traffic Engineering, Guilin University of Electronic Science and Technology, Guilin 541004, China; xzsj@mails.guet.edu.cn

**Keywords:** underwater image enhancement, degradation-aware classification, UNet, multi-stage fusion, adaptive fusion network, scene-specific enhancement

## Abstract

Underwater images frequently suffer from color casts, low illumination, and blur due to wavelength-dependent absorption and scattering. We present a practical two-stage, modular, and degradation-aware framework designed for real-time enhancement, prioritizing deployability on edge devices. Stage I employs a lightweight CNN to classify inputs into three dominant degradation classes (color cast, low light, blur) with 91.85% accuracy on an EUVP subset. Stage II applies three scene-specific lightweight enhancement pipelines and fuses their outputs using two alternative learnable modules: a global Linear Fusion and a LiteUNetFusion (spatially adaptive weighting with optional residual correction). Compared to the three single-scene optimizers (average PSNR = 19.0 dB; mean UCIQE ≈ 0.597; mean UIQM ≈ 2.07), the Linear Fusion improves PSNR by +2.6 dB on average and yields roughly +20.7% in UCIQE and +21.0% in UIQM, while maintaining low latency (~90 ms per 640 × 480 frame on an Intel i5-13400F (Intel Corporation, Santa Clara, CA, USA). The LiteUNetFusion further refines results: it raises PSNR by +1.5 dB over the Linear model (23.1 vs. 21.6 dB), brings modest perceptual gains (UCIQE from 0.72 to 0.74, UIQM 2.5 to 2.8) at a runtime of ≈125 ms per 640 × 480 frame, and better preserves local texture and color consistency in mixed-degradation scenes. We release implementation details for reproducibility and discuss limitations (e.g., occasional blur/noise amplification and domain generalization) together with future directions.

## 1. Introduction

Underwater imaging is crucial for tasks such as marine archeology, biological observation, and autonomous underwater vehicle navigation. However, due to wavelength-dependent light absorption and multipath scattering in turbid water [1], it still faces fundamental challenges, resulting in serious color shift, insufficient illumination, and image blurring, which in turn affects visual perception and downstream tasks [2]. This challenge is consistently highlighted in recent surveys [3,4]. It is critical to acknowledge that underwater degradations are seldom mutually exclusive; real-world scenes frequently exhibit compounded pathologies, such as color cast coupled with low illumination, or blur interwoven with haze. To maintain a tractable and efficient pipeline design, our framework adopts a three-class, hard-label classification scheme to identify the dominant degradation type. This pragmatic simplification facilitates the initial routing to specialized enhancement modules. However, we explicitly recognize the inherent limitations of this discrete modeling approach in handling complex, mixed-degradation scenarios, a challenge that is analytically addressed in our fusion stage and further discussed in the limitations and future work sections.

Underwater degradation is often a combination of low-level restoration problems such as defogging, denoising, deblurring, and low-light enhancement [5]. The existing methods mainly innovate from six aspects: network architecture, learning strategy, learning stage, auxiliary task, domain perspective, and disentanglement fusion [6,7,8]. In terms of network architecture, lightweight design has received extensive attention. Shallow-UWNet [9] reduces the number of parameters by compressing the network structure to achieve fast reasoning. U-Trans [10] uses a transformer architecture to capture long-distance dependencies. However, these methods are often difficult to balance between computational efficiency and enhancement quality. In terms of multi-stage methods, WaterNet [11] integrates multiple preprocessing results (white balance, histogram equalization, etc.) and learns weights through the gating mechanism. CUIE [12] adopts a coarse-to-fine two-stage enhancement strategy. Unlike end-to-end models that obscure intermediate reasoning, our framework adopts a modular, degradation-aware design that first classifies the dominant degradation type and then applies targeted enhancement. This modular decomposition into classification, enhancement, and fusion stages enhances system interpretability and facilitates lightweight, real-time deployment—a key requirement for edge-computing scenarios.

Although based on classical physics methods and histogram equalization methods (such as Retinex, CLAHE) [13,14], they are efficient and easy to explain, and can partially solve a degradation problem, but they lack robustness in different scenarios [11,15,16]; in contrast, end-to-end deep learning methods can adaptively degrade, but often sacrifice real-time performance or require a large amount of labeled data [17,18]. Representative methods include WaterNet, UColor, U-Trans, etc. [10,11,15]. Gao et al. and Panita et al. found that multi-band detail optimization and adaptive color correction can effectively solve the problems of low contrast, color distortion, and detail loss in underwater photography [19,20]. In addition, the data and evaluation itself also have significant limitations: the ‘clear’ reference of real-world pairing is difficult to obtain, and common benchmarks (such as UIEB, EUVP, etc.) use voting or domain conversion/synthesis strategies to generate reference images, which will introduce bias and affect the generalization evaluation of the method in real waters [11,21,22]. Therefore, high scores on synthetic data alone cannot prove robustness under all real-sea conditions. In practical engineering requirements, such as real-time enhancement on submersibles, unmanned boats, or embedded shooting devices, it is required to take into account interpretability, speed, and certain enhancement capabilities under limited computing resources [9,23,24], which are engineering tradeoffs in edge computing scenarios [25].

In order to bridge the gap between versatility and computational efficiency, and deal with multiple types of degradation conditions as much as possible under real-time available engineering constraints, our goal is to provide a practical solution that can be deployed on edge devices with speed, interpretability, and robustness. Therefore, the primary goal of this work is not to propose a new fundamental restoration model, but to design and validate a practical, lightweight, and interpretable framework that balances enhancement quality with computational efficiency for real-world deployment. Based on this, a two-stage degradation-aware lightweight multi-channel enhancement and learning fusion framework is proposed. The framework first uses a lightweight CNN to quickly estimate the dominant degradation category and output low-dimensional features, then applies special lightweight enhancement pipelines to the three types of degradation, and finally dynamically weights the three-way results through a global linear or spatial adaptive LiteUNet fuser. This framework is particularly suited for real-time applications on resource-constrained platforms, such as autonomous underwater vehicles or embedded imaging systems. Compared with the single scene optimizer, this method has a significant improvement in both objective indicators and subjective perception on the EUVP test set, achieving a PSNR gain of 3.2 dB, UCIQE/UIQM indicators are increased by 10%/8%, respectively, while maintaining real-time processing capabilities (each 640 × 480 image processing time on Intel i5-13400F CPU is less than 130 ms).

The main contributions of this work are summarized as follows, collectively forming a practical system for edge-oriented underwater image enhancement:

1. A practical, lightweight framework for edge deployment. We present a modular two-stage pipeline specifically designed for CPU-based, real-time underwater image enhancement, prioritizing deployability on resource-constrained devices.

2. Degradation-aware routing with a lightweight CNN. A compact CNN classifier achieves 91.85% accuracy in identifying the dominant degradation type (color cast, low light, blur) on EUVP, providing a crucial prior for routing images to targeted enhancement operators. Its intermediate features also serve as conditional inputs to guide the fusion stage.

3. Specialized, efficient enhancement operators. Three scene-specific, computationally efficient pipelines (e.g., CLAHE-based, Laplacian sharpening) are developed, each optimized for a primary degradation type. Their non-deep-learning nature ensures low computational cost and stability.

4. Learnable adaptive fusion strategies. We propose and compare two fusion schemes, a highly efficient global Linear Fusion and a more expressive LiteUNetFusion with spatial-adaptive weighting. These modules dynamically integrate the three pipeline outputs, with the LiteUNet variant demonstrating improved handling of degradation scenes.

## 2. Methodology

In this section, we present a practical two-stage, degradation-aware pipeline for underwater image enhancement [13]. The first stage performs coarse degradation estimation and produces either a low-dimensional feature vector or a spatial feature map used by the subsequent fusion module. The second stage applies three lightweight, scene-specific enhancement operators and fuses their outputs using a learnable fusion network that produces per-pixel or per-image weights. Below, we detail the degradation model, feature computation, classifier architecture, pipeline implementations, fusion architecture, and the training protocol, including losses and implementation specifics.

### 2.1. Dataset Selection

#### 2.1.1. EUVP Dataset

EUVP provides pairs of ‘degraded–clear’ images, which allows us to perform rigorous quantitative comparisons on pixel-level or reference metrics (such as PSNR, SSIM), rather than relying solely on no-reference metrics. This is crucial to verify the improvement of different enhancement strategies (single-scene optimizer, linear fusion, and LiteUNetFusion) on objective indicators. Secondly, EUVP contains examples from a variety of shooting conditions and synthesis parameters (including different degrees of color cast, low illumination, and scattering-induced blurring), covering the three types of degradation types we focus on, which is conducive to training and evaluating degradation classifiers and scene-specific pipelines. Third, the size of EUVP is moderate (thousands of paired images), which can not only support the training of deep models, but also facilitate manual verification and subjective subset evaluation (MOS), which is helpful for the comparison of subjective and objective indicators in the report.

From the EUVP paired dataset, each subset (underwater dark, underwater imagenet, underwater scenes) selects as many samples as possible with a uniform distribution of numbers and degradation types. Based on the selected clear EUVP images [17], using the Jaffe–McGlamery underwater imaging model, three typical degradation types were synthesized by sampling a physically reasonable parameter range (background light, scattering coefficient *β*, attenuation per channel), color cast (hue change), low light (V-channel energy reduction), and blur (scattering or low-pass transmittance increase). By sampling a physically reasonable parameter range (background light, scattering coefficient, attenuation per channel), 2500 datasets were obtained. In order to ensure that the synthesized category labels are consistent with human perception, we performed manual verification, and three independent annotators visually examined 2500 images each to correct the obvious mismatch between the synthesized labels and the perceptually dominant degradation. The final image label is determined by a majority vote; pictures without a majority of votes are jointly adjudicated. After verification, only a small number of samples need to be corrected, indicating that the degradation of physical simulation is highly consistent with human judgment. Table 1 summarizes the consistency between annotation statistics and ratings.

The ‘clear’ reference image in the EUVP dataset is generated by the synthesis of physical models, rather than the Ground Truth in real physical scenes. Although the Jaffe-McClamery model [15] can simulate the main characteristics of underwater light attenuation, this synthesis process may still introduce color and contrast preferences, which cannot fully cover the complex degradation mode of the real underwater environment [11]. In addition, real underwater images often contain a mixture of multiple degradation types, and synthetic data may be difficult to accurately simulate this mixing characteristic [21]. In order to evaluate the impact of this synthesis bias on our method, we use the real underwater image dataset UIEB for additional generalization verification in Section 5. The reference image of UIEB is generated by multi-method integration and manual voting, which represents a more realistic visual preference and can more comprehensively test the robustness of the model in real scenes.

#### 2.1.2. UIEB Dataset

In addition to the synthetic or semi-synthetic dataset EUVP, we also use UIEB for additional generalization verification in the follow-up work of the article, for the following reasons. First of all, UIEB contains a large number of scenes from real underwater shooting, and its ‘clear’ reference images are mostly generated by manual or multi-method integration, which represents the diversified visual preferences of real enhanced targets. Therefore, it has supplementary value in testing the generalization of the model to real-world scenes. Secondly, UIEB images are widely distributed in water color, visibility, and captured objects (corals, fish, sediments, etc.), which helps to discover the weaknesses of the model in the recovery of specific objects or details (such as fish color fidelity or weak texture recovery), thus providing a realistic test for the robustness evaluation of the method. Third, using UIEB as a cross-dataset validation set only for testing (held-out) can reveal the performance degradation and index offset caused by the synthesis bias of training datasets (such as EUVP), which is convenient for us to make an empirical explanation on GT bias, index applicability, and future domain adaptation work in the Discussion.

Based on the above reasons, we use UIEB as an important cross-dataset validation set (not involved in training). Detailed quantitative comparisons and representative visual results on UIEB are presented in Section 5 to more comprehensively evaluate the practicality and limitations of the method in real underwater scenes.

In addition to standard deterministic metrics, we evaluated the degradation perception module probabilistically. The classifier emits a per-sample softmax probability vector; we aggregate these into soft-confusion matrices (expected probability mass per true class), compute per-class calibration (expected calibration error, ECE), and plot reliability diagrams. To obtain interval-valued approximate membership (analogous to Type-2 fuzzy intervals), we used Monte Carlo Dropout (MC dropout) with T = 5 stochastic forward passes and report the mean ± standard deviation as a practical interval estimate. These probabilistic diagnostics were computed both in-distribution (EUVP held-out) and cross-dataset (UIEB-derived sets) to quantify generalization and to inform soft or interval conditioning for fusion. All results reported in Section 5.4 (including soft-confusion matrices, ECE values, and MC dropout intervals) are computed from the EUVP-trained classifier without re-training. Soft or interval-valued diagnostics are obtained by interpreting the classifier’s softmax outputs and by running MC dropout (T = 5) at inference; they are therefore post hoc analyses of the existing model rather than results of a newly trained “soft-label” classifier or a full Type-2 fuzzy system.

### 2.2. Physical Degradation Modeling

**Figure 1:**
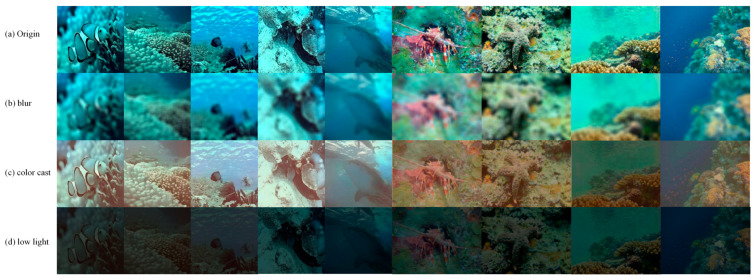
Example images illustrating the three targeted degradation types synthesized from the EUVP dataset: (**a**) Origin clear reference; (**b**) blur; (**c**) color cast; (**d**) low light.

As illustrated in Figure 1, these mechanisms manifest as three perceptually distinct degradations. We adopt the widely used Jaffe–McGlamery model to characterize underwater image degradation by light absorption and scattering [26,27]. In this model, the observed image I(x) is as follows:

(1) I(x)=J(x)×t(x)+B×(1−t(x))

where I(x) is a degraded image, J(x) is a clear image, and $t(x)=e^{-\beta d}$ means transmittance, medium-based *β,* and distance *d*, and *B* is background light intensity.

This single equation simultaneously captures wavelength-dependent color cast (through $e^{-\beta d}$) and both forward- and back-scattering effects (through the additive term involving *B*). We use this formulation to synthetically degrade clear EUVP images [22] for controlled experiments and to justify our feature-based classification approach.

In the current work, based on some pragmatic and scientific considerations, we select three categories of color cast, low light, and blur as the categories of marine image enhancement. Firstly, these three types correspond to different physical mechanisms in the Jaffe–McGlamery model, and wavelength-dependent attenuation corresponds to color cast. *V*-channel energy reduction corresponds to low light; scattering enhancement and transmission reduction correspond to blurring, and are widely mentioned in the literature review. Secondly, using our designed compact feature set (color channel imbalance, average brightness, Laplace variance) and lightweight CNN, these three categories are easy to distinguish in perception; when the number of categories increases, the boundary overlap increases significantly. For example, low light is easily accompanied by slight blurring, and the classification income decreases. Third, from the perspective of engineering implementation, the three-class partitioning method makes the efficient expert operator designed for each class cover the dominant problem and maintain a small fusion decision space, which is conducive to robustness and runtime delay control. We observed in the previous experiments that although the three-classification method will have a problem that some degradation types cannot be covered, if the category is subdivided into more subcategories, such as different types of color casts or finer-grained turbidity classification, it will bring label ambiguity and only marginal performance improvement.

Therefore, under the current consideration of balancing efficiency and effect, the three categories are used as a balanced and practical classification scheme. We recognize that real images often show mixed degradation types, and the inherent limitation of modeling the continuous mixing characteristics of underwater degradation in the real world is that the boundaries between these degradation categories (such as low illumination with slight blurring) are often blurred. The framework selects and weights the corresponding enhancement process by predicting the dominant category, and the subsequent fusion module (especially LiteUNetFusion) processes local changes and mixing effects through spatial adaptive weighting, allowing local adjustments to the enhanced output to solve this limitation. The use of more refined or continuous degradation representations (such as probability or fuzzy labels) in future work is a feasible direction to capture complex mixed effects.

### 2.3. Feature Extraction

Given an input RGB image *I*, we compute the following:

1. Color-cast score $f_{cc}$, convert *I* to channels R, G, B; compute *ColorCastScore*:

(2) $$\mu_{X}=mean_{X}=\frac{1}{N}\sum_{i=1}^{N}X_{i}$$

(3) $$ColorCastScore=\max\left(\begin{array}{l} \left|mean_{R}-mean_{G}\right|,\\ \left|mean_{G}-mean_{B}\right|,\\ \left|mean_{B}-mean_{R}\right| \end{array}\right)$$

where *X* means {R, G, B}, which are the red, green, and blue channels of the pixel, respectively; *N* is the total number of pixels.

2. Low-light score $f_{l}$, convert *I* to grayscale $I_{gray}$; compute the mean intensity $\mu_{gray}$:

(4) $$\mu_{gray}=\frac{1}{N}\sum_{i=1}^{N}I_{i}$$

where *I_i_* is the luminance value of each pixel in the gray scale map; the range of values is [0, 255].

3. Blur score $f_{b}$, apply the Laplacian operator $\nabla^{2}$ to $I_{gray}$ and take its variance

(5) $$\begin{array}{c} Var\left(\nabla^{2}I_{gray}\right)\\ ClarityScore=Var\left(\frac{\partial^{2}I}{\partial x^{2}}+\frac{\partial^{2}I}{\partial y^{2}}\right) \end{array}$$

where *I* is the gray scale map; the Laplacian operator reflects the edge strength of the image; a larger variance indicates a sharper image.

Each image is thus represented by the feature vector $f=\left[f_{cc},f_{l},f_{b}\right]$ [28]. Prior to clustering or CNN input, we standardize each feature dimension to zero mean and unit variance. Features are zero-mean and unit-variance standardized before use.

Before use, each feature dimension is standardized (zero-mean, unit-variance) using statistics computed on the training set. These features are used both for simple-rule or *K*-means baselines and as auxiliary inputs to the fusion network when precomputed feature maps are available.

### 2.4. Comparison of Classification Methods

In our framework, the classification network serves a dual functional role:

1. Dominant degradation labeling: It provides a discrete three-class label (color_cast, low_light, blur) to route the image to the appropriate scene-specific enhancement pipeline.

2. Conditional feature extraction: More importantly, its intermediate convolutional feature maps encode spatial degradation patterns and confidence distributions, which are upsampled and fed as conditional inputs to the fusion module. This enables the fusion network to perform spatially adaptive weighting beyond a simple one-hot label, thereby enhancing its capability to handle mixed and locally varying degradations.

We employ a lightweight convolutional neural network (CNN) as the degradation classifier in our framework, driven by its balanced capability in feature representation, conditional information provision, and computational efficiency. Unlike handcrafted features such as color statistics, average brightness, or Laplacian variance, which may suffice for simple degradations but struggle with mixed or complex underwater conditions, CNNs automatically learn hierarchical spatial and texture patterns. This enables robust discrimination among overlapping degradation types, such as low-light scenes with subtle blur or color cast. Moreover, the CNN’s intermediate feature maps serve as rich conditional inputs to the subsequent fusion module, conveying fine-grained spatial confidence and semantic cues beyond mere one-hot labels, thereby guiding more adaptive fusion. To meet real-time constraints on edge devices, our CNN is deliberately designed with only three convolutional layers, batch normalization, and global pooling, ensuring low inference latency while maintaining high accuracy. Empirical validation on the EUVP subset confirms that this lightweight CNN achieves 91.85% classification accuracy, significantly outperforming threshold-based rules (62%) and *K*-means clustering (71%), which justifies its role as a reliable and efficient degradation-aware component in our pipeline.

The preliminary model classification training and verification work mainly uses the EUVP dataset. In order to ensure the reliability and reproducibility of the classification results, we adopted strict control measures in the data division, training, and verification links. From 2500 samples, 500 were retained as an independent held-out test set (for final report and comparison), and the remaining 2000 were used for training and verification. The test set is not used in any model selection or hyperparameter search process to avoid information leakage. The 2000 training candidate sets were divided into a training set (1600) and a validation set (400) by 80%/20% using stratified split sampling to ensure that the proportion of each category in each subset was approximately the same, so as to avoid the deviation caused by category imbalance. All partitions are generated by fixed random seeds for reproduction and review. Manually proofread the currently used data, avoid the dataset source containing near-frame or multi-view repeated images, and calibrate the class imbalance of each classification to ensure the uniform distribution of each type of dataset. By using the early stop strategy, the accuracy or loss of the validation set is used as the monitoring target. If there is no improvement in several rounds, the training is stopped, and the model is rolled back to the best model, thereby reducing the overfitting of the validation set. Ensure that the test set is completely independent of any training and hyperparameter search process; hyperparameters are determined by grid or random search on the training or validation set, and the test set is only used for final evaluation.

#### 2.4.1. *K*-Means

As a lightweight and interpretable baseline, we use *K*-means clustering (*K* = 3) for unsupervised classification on the manual 3D feature space [f_colorcast, f_lowlight, f_blur] and compare it with a supervised CNN. The specific process is as follows: first, zero-mean or unit-variance standardization is performed on each feature dimension on the training set to avoid scale differences affecting Euclidean distance calculation; then, *K*-means is performed on the normalized three-dimensional vector, using multiple random initializations and selecting the minimum SSE result to reduce the initialization sensitivity. The mapping of clustering results to semantic labels (color_cast/low_light/blur) is achieved by using a majority vote on the validation set, that is, each cluster is assigned a semantic name to the real label with the highest frequency in the validation set.

*K*-means is characterized by low computational cost, strong interpretability, and fast rough division without labels. However, its limitations are also obvious; it only relies on global statistical features, ignores spatial and texture information, is sensitive to feature scale, initialization, and cluster shape, and cannot handle complex multi-modal distributions within the class. Therefore, we use *K*-means as a baseline to show the separability and limitations of manual features, and compare it with threshold rules to prove the advantages of lightweight CNN under complex or mixed degradation conditions.

#### 2.4.2. LightCNN

**Figure 2:**
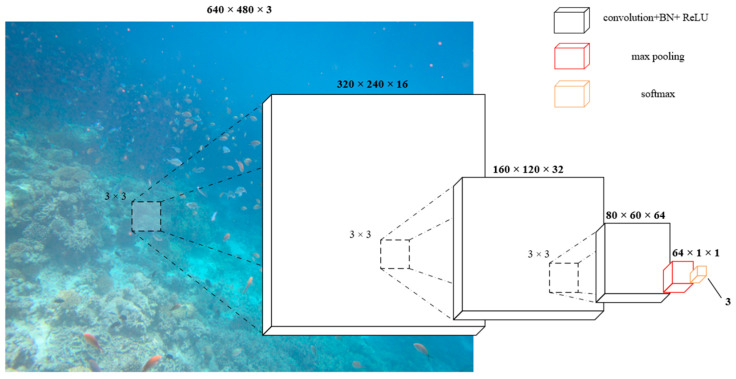
Architecture of the lightweight CNN (LightCNN) used for degradation classification. Notes: (1) The dashed lines indicate the 3 × 3 convolutional kernel sliding window (for feature extraction) and the dimension mapping relationship between adjacent layers; (2) The number labels (e.g., 640 × 480 × 3) represent the feature map size (width × height × channel number) of each layer; (3) The block styles correspond to operations: solid white block (convolution + BN + ReLU), red block (max pooling), orange block (softmax).

We design a lightweight convolutional classifier that accepts RGB images and outputs three types of degraded category logits to avoid the ‘black box’ problem of the end-to-end model, shown in Figure 2.

The backbone consists of three convolutional stages:

Conv1: Conv2d(3, 16, kernel = 3, stride = 2, padding = 1) + BN + ReLU.

Conv2: Conv2d(16, 32, kernel = 3, stride = 2, padding = 1) + BN + ReLU.

Conv3: Conv2d(32, FeatC, kernel = 3, stride = 2, padding = 1) + BN + ReLU.

An adaptive average pooling reduces spatial size to 1 × 1, followed by a fully connected layer producing 3 logits. The network also returns the Conv3 feature map (shape B × FeatC × H/8 × W/8) when requested; these feature maps may be upsampled and concatenated to the fusion network input. The classifier is trained on 2000 labeled images (80% train/20% val split within the classifier dataset) using cross-entropy loss.

The lightweight convolutional neural network (LightCNN) is selected instead of relying solely on manual features or simple clustering, for the following reasons. Although a convolutional neural network (CNN) is not a new technology, a large number of previous studies and reviews have repeatedly proved the effectiveness and robustness of CNN in image degradation or scene discrimination tasks [3,29]. Even if manual features such as color cast, brightness, and sharpness have strong discrimination in most cases, it is difficult for a single statistical feature to stably separate categories in the case of mixed degradation, such as slight blur and low light or local degradation at the same time. CNN can learn multi-level spatial and local texture features, and has stronger discrimination ability for complex mixed degradation. In addition to the output category probability, the penultimate layer or the last set of convolutional feature maps of CNN can be input into the fusion module as low-dimensional conditional vectors or spatial feature maps to guide global linear or spatial adaptive fusion. This feature-based conditional mechanism is superior to using only one-hot tags because it carries fine-grained information about ‘confidence’, ‘semantics’, and spatial distribution, and provides useful conditional features for fusion modules, which helps to improve fusion performance.

The innovation of this work is not to propose a new classifier structure, but to combine the proven lightweight CNN with three scene-specific lightweight enhancement pipelines and two types of learnable fuses into a modular, engineering-oriented deployment system. The CNN we designed is shallow, with only a small number of convolution channels and levels, supplemented by lightweight structures such as batch normalization and global pooling, and low training and reasoning overhead; it can also achieve low latency on ordinary CPU or embedded platforms. The measured classification time in this paper is less than 25 ms, which is lightweight and cost-controllable, easy to deploy at the edge, and meets the needs of engineering landing.

CNN has robustness and verifiability. In our subsequent comparative experiments, the lightweight CNN significantly exceeds the three-dimensional feature clustering based on threshold rules or *K*-means while retaining real-time performance, and its prediction behavior can be quantitatively verified by conventional model diagnostic methods such as confusion matrix and ROC/AUC, so as to improve repeatability and interpretability.

#### 2.4.3. Classification Result

**Figure 3:**
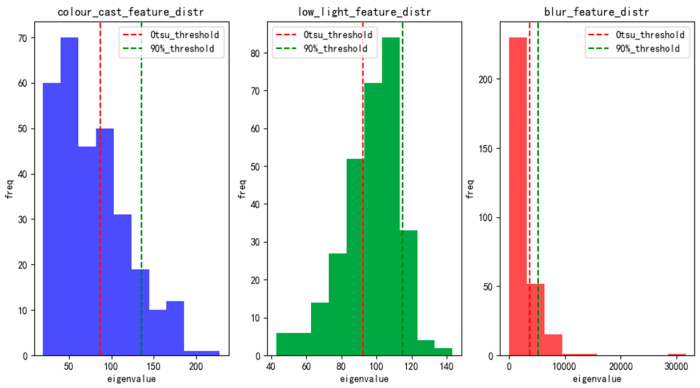
Feature histograms for three degradation classes, with Otsu thresholds indicated.

We compare three classifiers on a held-out test set of 500 EUVP images. Threshold-based rules include applying Otsu’s method independently to each feature, which yields 62% overall accuracy, with most samples misclassified as color cast. Figure 3 displays histograms of $f_{cc},f_{l},f_{b}$, showing that color-cast, low-light, and blur samples occupy largely non-overlapping intervals. *K*-means clustering (*K* = 3): clustering the three-dimensional feature vectors achieves 71% accuracy (silhouette = 0.34), but boundaries between low-light and blur remain fuzzy.

This group of images shows the eigenvalue histograms of three types of underwater degradation, including color cast, low light, and blur, and the results of Otsu and 90% double threshold classification. Although there are some differences in the feature distribution of the three degradations, the dual-threshold can initially segment the degraded and normal samples, and has a certain classification ability, but the histogram can still be seen in the feature overlap (such as the low light and the color deviation feature value range crossed), the threshold method is difficult to accurately identify the edge samples (such as fuzzy samples close to the threshold are easy to misjudge) or mixed degradation scenes (such as images with both color deviation and low light), the overall classification effect is not ideal, and it fails to fully meet the high requirements of degradation perception for classification accuracy.

**Figure 4:**
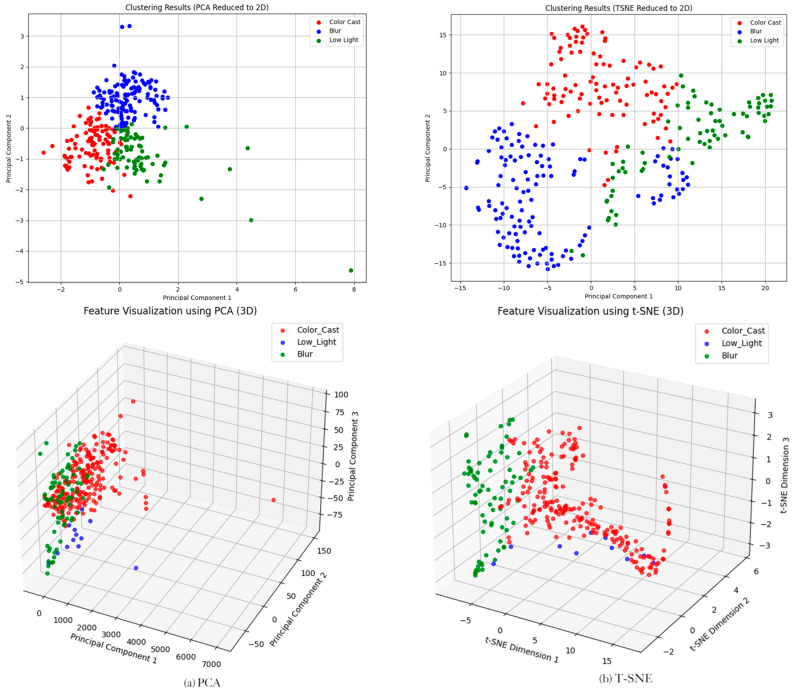
*K*-means clustering results (*K* = 3) visualized via PCA and t-SNE. (**a**) Combined visualization of PCA results: upper subplot is the 2D clustering result (high-dimensional features reduced to 2D via PCA), lower subplot is the 3D feature visualization (high-dimensional features reduced to 3D via PCA); (**b**) Combined visualization of t-SNE results: upper subplot is the 2D clustering result (high-dimensional features reduced to 2D via t-SNE), lower subplot is the 3D feature visualization (high-dimensional features reduced to 3D via t-SNE).

Figure 4 shows the *K*-means clustering results based on 3D hand-designed features. The high-dimensional features are reduced to a two-dimensional space visualization by PCA and t-SNE, and the clustering quality statistics (square sum error, SSE, and contour coefficient) are attached. From the scatter plot after dimensionality reduction, the clustering boundaries of the three types of samples are blurred, especially the low-light (red) and fuzzy (green) samples, which have significant overlap, and a large number of red and green points are mixed, failing to form independent dense clusters. Although the color-biased (blue) samples are relatively concentrated, some points are still scattered in other clusters. The clustering quality statistics further confirm this conclusion: the contour coefficient is about 0.34, indicating that the clustering cohesion is medium (the similarity of samples in the cluster is general), while the higher SSE value (combined with visual speculation) indicates that the samples in the cluster are more dispersed.

**Figure 5:**
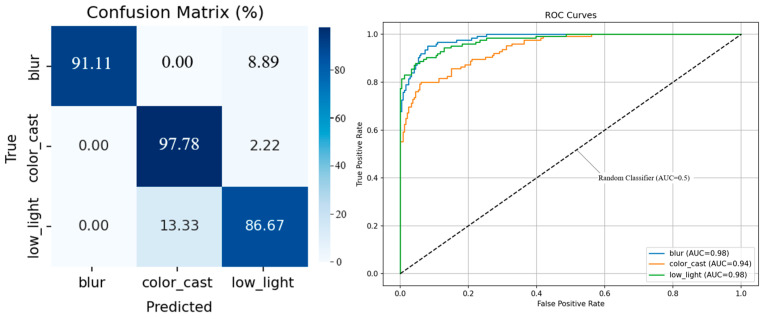
Performance of the LightCNN classifier on the held-out test set: (**left**) normalized confusion matrix (in percent); (**right**) ROC curves with AUC values for each class.

Figure 5 shows the confusion matrix on the retained test set, showing the count ratio of each class; the ROC curve and AUC of each degradation category; the training and verification accuracy, and the loss curve with the change in training rounds. The main results are as follows: 1. The overall accuracy rate is 91.85%. 2. Most of the classification errors occur between low-light and color cast categories, which indicates the complexity of underwater image enhancement, and there is a combination of multiple degradation types. 3. The ROC-AUC values were more than 0.96, indicating that the average separability was strong. We use 128-dimensional reciprocal second-level features (rather than just hard labels) as the conditional input of the fusion module.

Compared with the classification results of the convolutional neural network (CNN) in Figure 5, the *K*-means clustering separation effect based on manual features is significantly worse. CNN can more accurately divide the three types of degraded boundaries with high accuracy. The F1 score is 0.86, and the ROC-AUC of each category is close to perfect. However, *K*-means has limited ability to express degradation types due to manual features, and cannot effectively distinguish similar degradation scenarios such as low light and blur, resulting in suboptimal clustering results. Based on the above considerations and their robustness and real-time inference costs, we use a lightweight CNN as the discriminator of the first stage, and use the intermediate features of the CNN as the conditional input of the fuser for joint experiments.

## 3. Scene-Specific Enhancement

In this section, we present three dedicated enhancement pipelines tailored to the three degradation types (color cast, low light, and blur). Each pipeline comprises a sequence of lightweight operations designed for real-time execution. We then evaluate each pipeline individually on its target degradation to demonstrate its effectiveness before fusion.

Color-Cast Enhancement: CLAHE + Gamma + Sharpening; CLAHE in LAB space, convert the input image *I* from BGR to CIELAB color space, yielding channels *L*, *A*, *B*. Apply contrast-limited adaptive histogram equalization (CLAHE) to the *L* channel:

(6) $$L^{\prime }=CLAHE(L;clipLimit=2.0,tileGridSize=(8,8))$$

Merge $L^{\prime }$ with the original *A*, *B*, and convert back to BGR.

To correct gamma, adjust brightness nonlinearly to compensate residual color bias:

(7) $$I_{\gamma }(x,y)=255\times {\left(\frac{I_{norm}(x,y)}{255}\right)}^{\gamma },\;\;\gamma =1.2$$

where $I_{norm}$ is the CLAHE output, and γ>1 brightens mid-tones.

And unsharp masking, compute a blurred version $I_{blur}=G_{\sigma =1.0}\times I_{\gamma }$

Enhance edges by the following:

(8) $$I_{sharp}=I_{\gamma }+\alpha \left(I_{\gamma }-I_{blur}\right),\;\;\alpha =1.0$$

This amplifies high-frequency details to restore crispness.

Low-Light Enhancement: HSV-CLAHE

Conversion to HSV, transform I to HSV space, obtaining *H*, *S*, *V* channels.

CLAHE on Value Channel: Apply CLAHE directly to the *V* channel:

(9) $$V^{\prime }=CLAHE(V;clipLimit=3.0,tileGridSize-(8,8))$$

Reconstruction: Recombine *H*, *S*, *V*′, and convert back to BGR.

This pipeline enhances local contrast in dark regions without distorting hue or saturation.

Blur Enhancement: Laplacian Sharpening

Laplacian Filter: Convolve the grayscale version of III with the Laplacian kernel:

(10) $$K=\left[\begin{array}{ccc} 0 & -1 & 0\\ -1 & 5 & -1\\ 0 & -1 & 0 \end{array}\right],\;\;L=K\times I_{gray}$$

Edge Reinforcement: Add the Laplacian response back to each channel of the original image:

(11) $$I_{sharp}=I+\beta stack\left(L,L,L\right),\;\;\beta =0.5$$

This emphasizes edges and mitigates the effect of scattering-induced blur.

To quantify each pipeline’s performance on its target degradation, we conduct individual pipeline validation experiments on matched subsets of the EUVP dataset. Color-cast pipeline notably boosts PSNR and UCIQE by restoring global contrast and neutralizing chromatic bias. Low-light pipeline achieves significant UIQM gains by enhancing local brightness without hue shifts. Blur pipeline improves structural similarity (SSIM) and UIQM by reinforcing edge definitions. These results confirm that each specialized pipeline effectively addresses its target degradation, laying the foundation for our adaptive fusion in the next Section.

**Figure 6:**
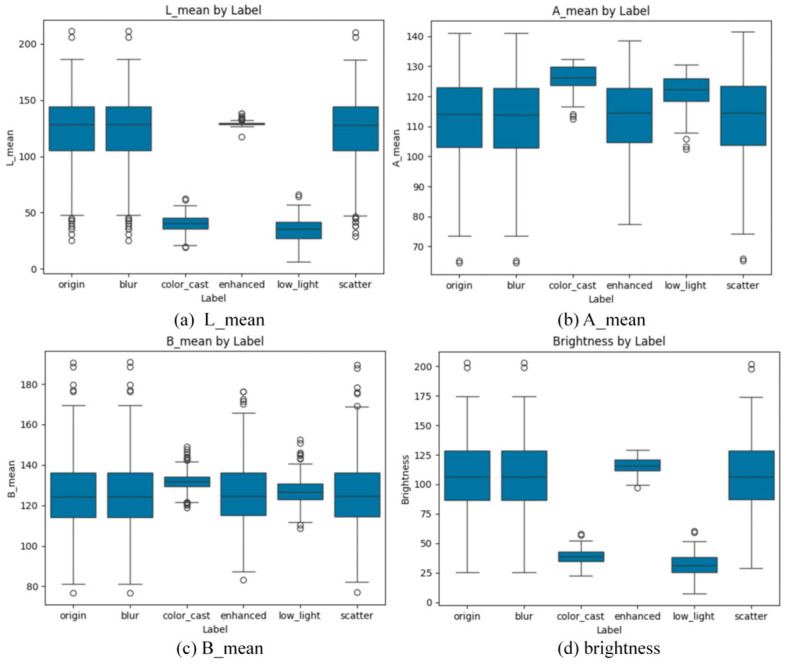
Distribution of color channel statistics (L, A, B) and brightness across original, degraded, and enhanced images. (**a**) Distribution of the mean value of the L color channel (L_mean) across different image types (original, blur, color_cast, enhanced, low_light, scatter); (**b**) Distribution of the mean value of the A color channel (A_mean) across different image types; (**c**) Distribution of the mean value of the B color channel (B_mean) across different image types; (**d**) Distribution of brightness across different image types. Circles in subplots denote boxplot outliers (data points beyond 1.5 × IQR), corresponding to extreme color/brightness values deviating from most data under the same image type.

Table 2 shows the performance of the single-scenario enhancement pipeline on the matching degradation subset (the value is the mean ± standard deviation of the subset). Each line reports the indicators before and after the application of the degradation dedicated pipeline. Columns are as follows: PSNR (dB), UCIQE, UIQM. The table shows that each pipeline has the greatest improvement in its target degradation (for example, the color-biased pipeline has PSNR increased by 2.3 dB, UCIQE increased by 20%). It should be noted that the single-scenario pipeline may degrade other degraded non-target indicators, which promotes the need for adaptive fusion. Figure 6 presents corresponding box plots, confirming that each degradation type is concentrated in a distinct feature range suitable for classification.

The box plot comprehensively shows the distribution characteristics of the original image, the three main degradation types (color cast, low light, blur), the scattering and special enhanced images in L, A, B color channels mean (L_mean, A_mean, B_mean), and brightness. In the L channel (Figure 6a), the median L_mean of the low-light image is significantly lower than that of the original image and other degraded types. After the individual pipeline enhancement, the L_mean is greatly improved, which is equivalent to the level of the original image, clearly reflecting the effective improvement of the brightness channel of the low-light image by the enhancement method. The L_mean of the color-cast and blurred image is less different from the original image, and there is no significant change after enhancement.

In channel A (Figure 6b), the median A_mean of the color-cast image is significantly higher than that of the original image, reflecting the deviation of the red–green difference. After enhancement, the A_mean falls back to the median range of the original image, which directly verifies the effect of color cast correction. The A_mean box-shaped whiskers of the scattering image are longer, indicating that the value fluctuates greatly. After enhancement, the whiskers are shortened (about 105–128), and the fluctuation is significantly reduced.

In channel B (Figure 6c), the median B_mean of the color-cast image is lower than that of the original image (the original image is about 122, and the color cast is about 130), indicating that there is a deviation in the blue–yellow color difference. After enhancement, the B_mean is restored to the median level of the original image (about 123), which further consolidates the effectiveness of color cast correction. The B_mean of low-light and blurred images is not much different from that of the original image, and the change is not obvious after enhancement.

The trend of the brightness index (Figure 6d) is highly consistent with the L channel: the median brightness of the low-light image is the lowest (about 27), and the brightness is significantly improved after enhancement, which is close to the normal level of the original image; the brightness of other degradation types (such as color cast, blur) is less different from the original image, and there is no significant change after enhancement. Overall, the enhancement method realizes directional optimization for the core features of different degradation types. It focuses on improving the brightness and L-channel value of low-light images, accurately corrects the color difference deviation of A and B channels for color-biased images, and effectively reduces the fluctuation of channel values for scattering images. Finally, the distribution of each channel of the enhanced image is closer to the normal level of the original image, which reflects that the enhancement method has certain adaptability to underwater multi-degradation scenes.

**Figure 7:**
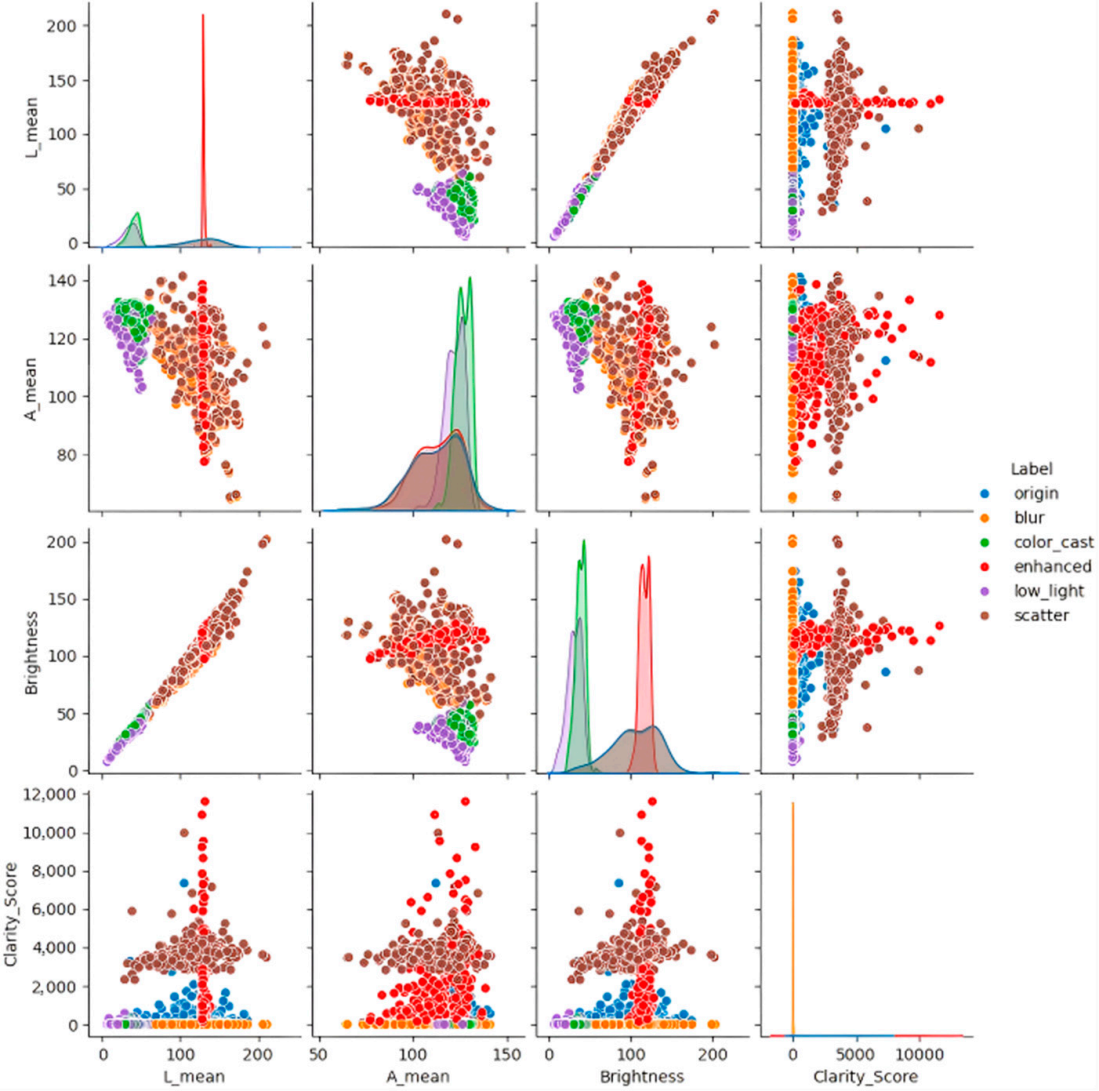
Pairwise scatter plots and histograms visualizing the relationships between L-mean, A-mean, brightness, and clarity score across different image groups on the EUVP subset. Colors in the plot correspond to image types (matching the legend: blue = origin, orange = blur, green = color_cast, red = Single channel enhanced, purple = low_light, brown = scatter).

Figure 7 shows the set of multi-subgraph visualization comprehensively presents the original image and three core degradation types (blur, color_cast, the statistical distribution and correlation characteristics of low_light), catter, and enhanced images on L-channel mean (L_mean), A-channel mean (A_mean), and brightness, as well as clarity score.

From the perspective of L_mean (the first line), the scatter points of the low-light image (purple) are concentrated in the low-value range of 0–50, and the histogram peak position is obviously shifted to the left (median about 30), which is much lower than the original image (blue, median about 150); after enhancement (red), the L_mean scatter points of the low-light image significantly move to the high-value area (the median rises to about 120), and the histogram peak position has a high degree of overlap with the original image, which clearly reflects the effective restoration of the low-light scene brightness channel by the enhancement method.

In A_mean (second row), the scatter distribution of the color-cast image (green) deviates from the original image (blue is concentrated in 100–150), and the histogram presents double peaks (about 80 and 140), reflecting the red–green difference anomaly; after enhancement (red), the A_mean scatter points of the color cast image converge to the concentrated area of the original image (the median is about 120), and the histogram peak position basically coincides with the original image, which directly verifies the effect of color-cast correction. The trend of brightness is highly consistent with L_mean: the brightness scatter of the low-light image (purple) is concentrated at 0–50, and after enhancement (red) rises to 100–150, which is close to the normal level of the original image (median is about 150). The brightness of the blurred (orange) and scattered (brown) images is slightly different from the original image, and there is no significant change after enhancement.

The scatter plot and correlation analysis of clarity score (fourth line) showed that the sharpness score of the original image (blue) was concentrated in 0–3500, and the scores of blurred (orange) and scattered (brown) images were significantly lower (mostly in 0–500). After enhancement (red), the clarity score of blurred and scattered images is improved to a certain extent, and the distribution range is more concentrated; the sharpness score of the enhanced low-light image (purple) is also improved, which further confirms the improvement of image detail and contrast by the enhancement method. On the whole, the distribution of the enhanced image (red) on each index is close to the normal level of the original image (blue), the brightness and L-channel value of the low-light scene are significantly improved, the A-channel color difference in the color-biased scene is corrected, and the clarity score of the blurred and scattered scene is significantly improved. This directional optimization feature distribution intuitively verifies the adaptability and effectiveness of the enhancement method for different types of underwater degradation.

## 4. Adaptive Fusion Network

In this section, we introduce our learnable fusion module that dynamically blends the three scene-specific enhancement outputs based on the estimated degradation severity. By leveraging the intermediate features from our degradation classifier, the fusion network assigns optimal weights to each pipeline, yielding a single, high-quality enhanced image.

### 4.1. Fusion Model Architecture

#### 4.1.1. Linear Model

Let $O_{cc},O_{l},O_{b}$ denote the RGB outputs of the color-cast, low-light, and blur pipelines, respectively. We first extract the penultimate feature vector $h\in {\mathbb{R}}^{d}$ from the lightweight CNN classifier mentioned before, which encodes the network’s confidence in each degradation type. The fusion network consists of the following:

Fully Connected Layer:

(12) $$z=Wh+b,W\in {\mathbb{R}}^{3\times d},b\in {\mathbb{R}}^{d}$$

Softmax Weighting:

(13) $$\alpha =soft\max(z),\;\;\alpha_{i}=\frac{\exp(z_{i})}{\sum_{j=1}^{3}\exp(z_{i})},\;\;i\in \left\{cc,l,b\right\}$$

Weighted Summation:

(14) $$I_{out}=\alpha_{cc}O_{cc}+\alpha_{l}O_{l},\alpha_{b}O_{b}$$

This architecture requires only a single linear layer plus softmax, ensuring minimal overhead.

#### 4.1.2. LiteUNet Model

The linear fusion strategy may not dynamically adjust the contribution of the pipeline for the degradation types of different regions—such as background blur and foreground color cast—resulting in fusions that either overcorrect (such as foreground color oversaturation) or provide incomplete repair (such as unresolved background blur). To this end, this paper also proposes and tries a U-Net fusion model, which realizes the dynamic integration of multiple pipelines through the spatial adaptive weight map, and solves the degradation problem in different regions.

We also design a lightweight U-Net style fusion model (LiteUNetFusion) with an input channel number of inches (default is 12, including degradation features and output of three pipes). The number of basic channels of the model is set to base inch (default 24), and the model size can be adjusted according to demand.

The main structure of the model is as follows:

1. **Encoder**: The initial convolution block maps the input to the number of basic channels. Two downsampling blocks are used to gradually compress the spatial size and increase the number of channels.
2. **Bottleneck layer**: Two convolution operations are performed on high-level features to further extract abstract information.
3. **Decoder**: Two up-sampling blocks are adopted; each block restores resolution via bilinear interpolation or transposed convolution, and concatenates with the corresponding features of the encoder to preserve details.
4. **Output layer**: The weight logits are generated by a 1 × 1 convolution. If predict_residual is enabled, an additional residual tensor is output through the tanh activation function to optimize the final fusion result.

The forward propagation process of the model is as follows: the input passes through the encoder to obtain multi-level features; after being processed by the bottleneck layer, the features enter the decoder and gradually reconstruct the high-resolution weight map.

For a given input image, let *O*_1_, *O*_2_, and *O*_3_ represent the RGB outputs of color deviation, low illumination, and blur enhancement channels, respectively. First, the model generates pixel-level weights $\log\mathrm{its}Z\in {\mathbb{R}}^{N\times 3\times H\times W}$, then calculate the normalized weight map:

(15) $$w_{i}=\frac{\exp(Z_{i})}{\sum_{j=1}^{3}\exp(Z_{j})}$$

In order to further improve the quality, we introduce the residual correction term R, which is constrained in the range of [−1, 1] by the tanh activation function R=tanh(Conv(D)), where D is the final feature of the decoder, $R\in {\mathbb{R}}^{N\times 3\times H\times W}$. the complete fusion expression is

(16) $$F_{\mathrm{fused}}=\sum_{i=1}^{3}w_{i}O_{i}+\delta R$$

where *w_i_* is the normalized weight obtained by Softmax, R is the residual adjustment term, and the scaling factor *δ* is defaulted to 0.2.

**Figure 8:**
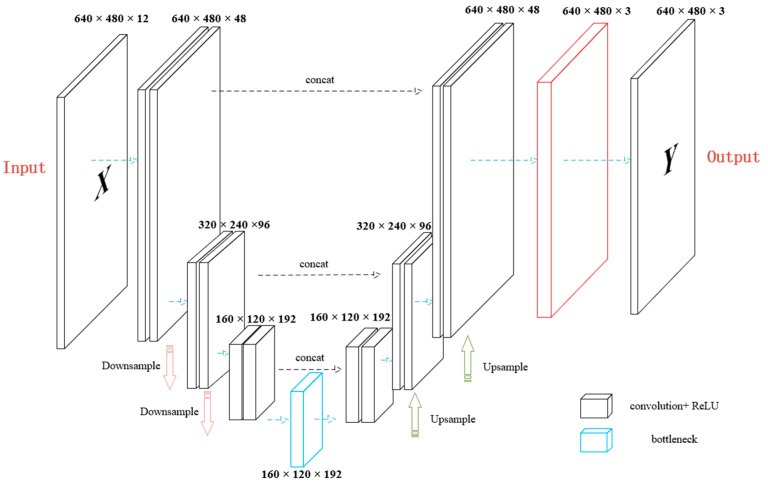
Architecture of the LiteUNetFusion module. Regular layer-wise arrows (the short dashed arrows, e.g., between 640 × 480 × 48 → 320 × 240 × 96, or 160 × 120 × 192 → 320 × 240 × 96): Represent feature transfer between adjacent layers in the same module (encoder downsampling process/decoder upsampling process). "concat"-labeled dashed arrows (the long dashed arrows): Represent U-Net skip connections—splicing features from the corresponding layer of the encoder (e.g., 320 × 240 × 96 in the encoder) with the upsampled features of the decoder, to retain high-resolution details.

Figure 8 shows the structure of LiteUNetFusion. The input part consists of two parts. One is the channel dimension splicing of three upstream pipeline outputs (E1, E2, E3). Each pipeline outputs a feature map with the same spatial size (H × W) (if each is 4 channels, the total number of channels is 12), and the multi-pipeline features are integrated into a unified input through splicing. Secondly, the optional spatial classifier features (such as a semantic category probability map) can inject additional semantic information according to the task requirements to further enhance the pertinence of fusion (such as emphasizing the fusion weight of edge or texture region). This input design ensures that the model can make full use of the complementary information of multiple pipelines while retaining flexible scalability.

The input to LiteUNetFusion concatenates the outputs of the three enhancement pipelines. Critically, it also incorporates the intermediate spatial feature maps from the LightCNN classifier (after upsampling). These features encode the network’s confidence distribution and contextual information across the image, providing a fine-grained, spatially varying prior that guides the fusion network beyond the simple global degradation label.

The encoder consists of two convolution down-sampling modules, and the core function is multi-scale feature extraction. Each module consists of two sets of convolutional layers (3 × 3 kernel, stride = 1, padding = 1, keeping the space size unchanged), batch normalization, and ReLU activation. Then, the space size is halved, and the number of channels is doubled by maximum pooling (2 × 2 kernel, stride = 2) (for example, when base_ch = 24, the number of output channels of the first module increases from 24 to 48). This combination of ‘convolution + pooling’ gradually pushes features from local details (shallow layers, such as edges, textures) to global contexts (deep layers, such as object structures, scene semantics), providing multi-scale feature support from fine to coarse for subsequent fusion, avoiding the limitations of single-scale features.

The decoder corresponds to two upsampling modules with skip connections, which aim to restore the spatial size and fuse multi-scale features. Each module uses bilinear interpolation (rather than transposed convolution, avoiding the checkerboard effect) to restore the spatial size of the feature map to the same size as the corresponding layer of the encoder (such as from H/4 × W/4 to H/2 × W/2), and then performs channel splicing with the feature map retained by the encoder (through jump connection, such as the output of the bottleneck layer is spliced with the feature of the second down-sampling layer of the encoder, and the number of channels is increased from 96 to 192), fuses global context and local details, and then adjusts the number of channels through the convolution layer. This design preserves the shallow-layer details extracted by the encoder through the skip connection, avoids the loss of information during the upsampling process of the decoder, and ensures that the final output map and the input remain the same size (H × W) to meet the needs of pixel-by-pixel processing.

The output head is the core component of the model to realize spatial adaptive fusion and residual correction. It contains two functional branches. One is the pixel-by-pixel weight map branch. The feature map (base_ch channel) output by the decoder is mapped to 3-channel logits by 1 × 1 convolution. After Softmax activation between channels, the three weights (w1, w2, w3) of each pixel are obtained, which satisfy w1 + w2 + w3 = 1. Each pixel value of the fused image is calculated by the weighted sum of the output of the corresponding pipeline. This pixel-by-pixel weight adjustment method allows the model to flexibly assign fusion weights according to local features (such as texture, edge strength), which is more suitable for complex scenes than fixed-weight global fusion. The second is an optional residual correction branch. If it is turned on, a bounded residual (range [−1, 1]) is generated by a 1 × 1 convolution and tanh activation, which is adjusted by the scaling factor δ and added to the fused image. The design of the residual term aims to locally correct the fusion artifacts. In the fusion process, problems such as blurring and color offset may occur due to the weight distribution deviation. The bounded residual fine-grained corrects these artifacts by small-scale adjustment (such as [−0.1, 0.1]), which does not destroy the overall structure of the fusion result and can improve the output quality. This design realizes spatial adaptive fusion and allows local correction of residual artifacts.

### 4.2. Training Strategy

**Figure 9:**
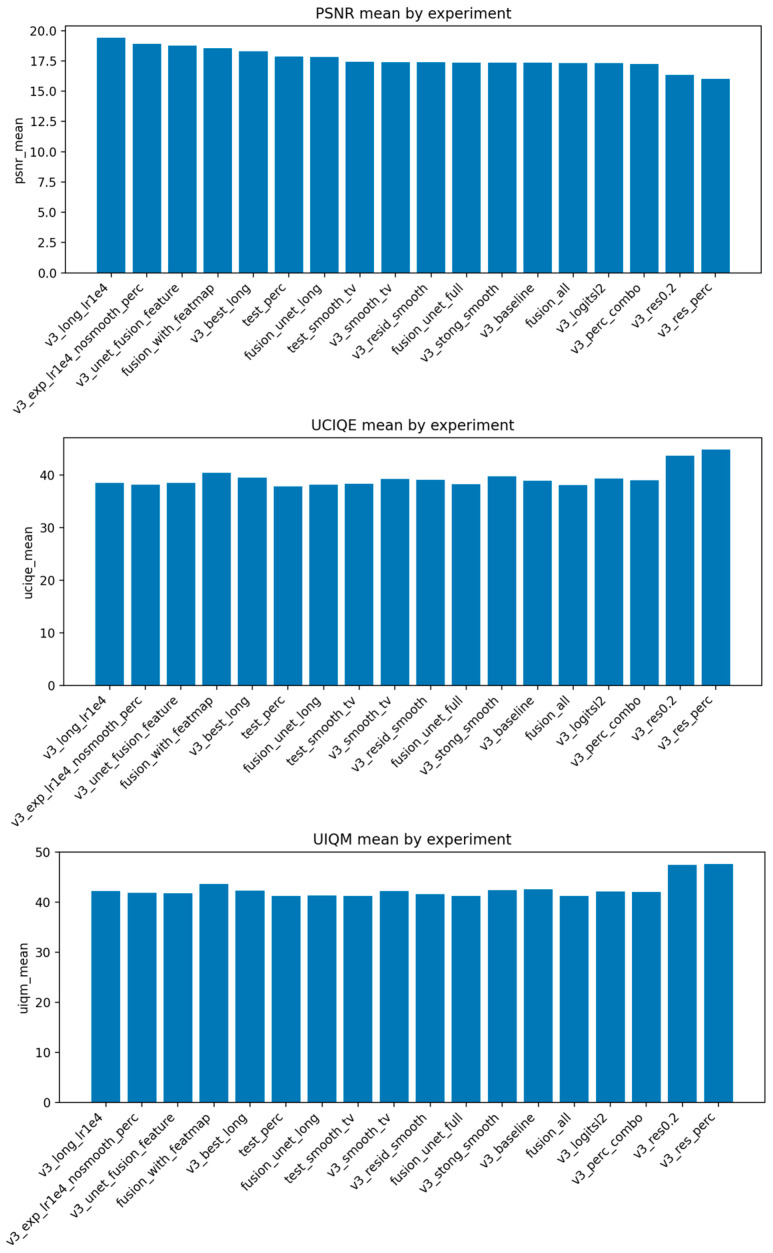
Hyperparameter search results for LiteUNetFusion (mean PSNR, UCIQE, UIQM).

Before the formal training of the subsequent model, we fixed the number of rounds and image pixels through grid search, tested the combination of parameters such as lambda_tv and perc_weight (a total of 18 sets of experiments), and determined the optimal range of each parameter. The experimental results are shown in Figure 9.

The pictures show the change trend of the model performance under the parameter combination of different experiments, which provides a reliable empirical basis for the parameter setting of subsequent formal training.

#### 4.2.1. Linear Strategy

Data: We use the same 2500 EUVP images, with clear references.

Linear Loss Function L:

(17) $$L=\underset{Pixel\;Loss}{\underset{︸}{∥I_{out}\;-I_{gt}\;{∥}_{1}}}+\lambda_{per}\;\underset{Perceptual\;Loss}{\underset{︸}{∥\phi (I_{out})-\phi (I_{gt}){∥}_{2}^{2}}}\;\;\;$$

where ϕ(·) extracts VGG-19 features and $\lambda_{per}$ = 0.01.

Optimization: Train the fusion layer parameters (*W*, *b*) for 30 epochs using Adam with learning rate 1 × 10^−4^.

During training, the CNN classifier’s weights remain frozen to preserve its degradation estimation performance.

#### 4.2.2. LiteUNet Strategy

LiteUNet multi-term loss function

(18) $$L=\lambda_{1}L_{l1}+\lambda_{p}L_{perc}+\lambda_{tv}L_{tv}+\lambda_{2}L_{l2}\;\;\;$$

Specifically, L_l1_: Mean absolute error between prediction and ground truth

(19) $$L_{l1}=\frac{1}{NCHW}\sum_{n=1}^{N}\sum_{c=1}^{3}\sum_{i=1}^{H}\sum_{j=1}^{W}\left|\left.{\hat{J}}_{n,c,i,j}-J_{n,c,i,j}\right|\right.\;\;\;$$

where $\hat{J}$ is the model output (the fused RGB image), and *J* is the clear image.

L_perc_: VGG-19 perceptual loss for feature-level alignment

(20) $$L_{\mathrm{perc}}=\sum_{l\in S}\frac{1}{n_{l}}\left\|\left.\phi_{l}\left(\hat{J}\right)-\phi_{l}\left(J\right)\right\|\right.$$

where $\phi_{l}(\cdot )$ represents the feature map extracted by VGG in the lth layer, and $n_{l}$ is the number of feature elements in this layer (which can be used for standardization).

L_tv_: Total variation regularization on weight maps to enforce spatial coherence

(21) $$L_{\mathrm{tv}}\left(W\right)=\frac{1}{N\cdot C\cdot \left(H-1\right)\cdot W}\sum_{n,c}\sum_{i=1}^{H-1}\sum_{j=1}^{W}\left|\left.W_{n,c,i+1,j}-W_{n,c,i,j}\right|\right.+\frac{1}{N\cdot C\cdot H\cdot \left(W-1\right)}\sum_{n,c}\sum_{i=1}^{H-1}\sum_{j=1}^{W}\left|\left.W_{n,c,i,j+1}-W_{n,c,i,j}\right|\right.$$

where $W\in {\mathbb{R}}^{N\times C\times H\times W}$ is the weighted graph normalized by softmax.

L_l2_: L2 penalty on weight logits to prevent extreme values

(22) $$L_{l2}=\frac{1}{\Omega }\sum_{w\in \Omega }z_{w}^{2}$$

where *z_w_* denotes the set of all logits elements, Ω denotes the number of elements.

### 4.3. Inference and Complexity

Based on the set of results generated for the single-condition enhancement model and the set of results for the combined model, it can be seen that the single condition generates a slightly better visual result for the picture, while the combined enhancement model performs well on the metrics. Below are some examples of the comparison of the picture effect.

#### 4.3.1. Linear Stratery Result

**Figure 10:**
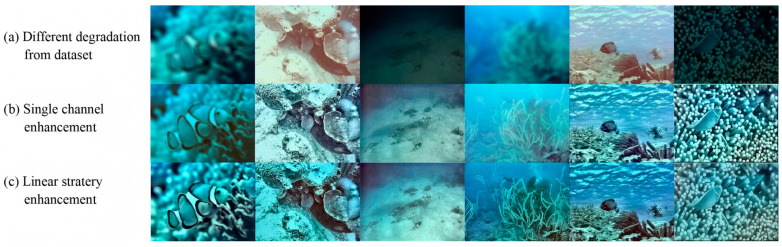
Visual comparison of enhancement results on EUVP images using single-pipeline strategies (color cast, low light, blur) and the Linear Fusion strategy. (**a**) Degraded samples from the dataset, (**b**) Enhancement results of single-pipeline strategies (included color cast, low light, blur), and (**c**) Enhancement results of the Linear Fusion strategy.

Figure 10 intuitively shows the application effect and advantages of the linear fusion strategy in underwater image enhancement tasks. By comparing the degraded image, the single enhanced pipeline output result, and the linear fusion result, the ability of the linear strategy to integrate multi-source information is clearly presented.

The single-channel strategy adopts customized lightweight operation for each degradation type. The color-biased image balances the L-channel through CLAHE in LAB space, Gamma correction compensates for color deviation and sharpens the enhanced details, and the color recovery is more balanced. The V-channel of the low-light image is processed by CLAHE in HSV space, and the dark contrast is improved, and the hue is not distorted. The blurred image is sharpened by Laplace sharpening to strengthen the edge, and the clarity is improved. These results verify the effectiveness of the single-channel strategy for target degradation, but also expose its limitations. It is only optimized for single degradation and has insufficient adaptability to complex scenarios (such as mixed degradation).

The linear strategy combines the output of three single-channel strategies. The results show that the linear strategy has a more comprehensive enhancement effect, the color cast image is further corrected, the dark details of the low-light image are richer, the edges of the blurred image are sharper, and the overall color, brightness, and clarity are better balanced. For example, the coral color in the blurred image is closer to the real, the seabed texture in the low-light image is clearer, and the edge of the fish in the blurred image is distinct. However, the disadvantage of the linear strategy is also obvious. Although it is better than the single-channel effect, the defects of the image are still obvious; the object is rough, the calibration is stiff, and there are some problems of excessive correction or detail loss.

This comparison not only proves the effectiveness of the single-channel strategy as the basic module, but also reflects the improvement of the comprehensive enhancement effect of the linear fusion strategy by integrating the advantages of multiple strategies. The results of the linear strategy are closer to the visual needs of the real underwater scene, and also imply the potential of the ‘degradation perception + multi-strategy fusion’ idea. Through the combination of targeted processing and global optimization, more robust underwater image enhancement can be achieved.

**Figure 11:**
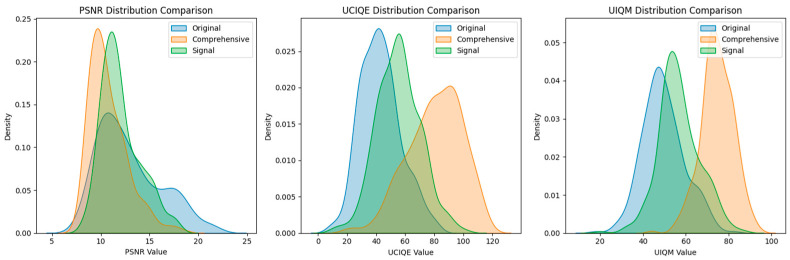
Density histograms comparing the distribution of PSNR, UCIQE, and UIQM scores between single-pipeline enhancements and the Linear Fusion strategy on the EUVP test set.

From the index of Figure 11, through the weighted combination of multi-source enhancement results, the linear fusion strategy seems to effectively suppress the limitations of a single method, while retaining the advantages of each special processing, thereby achieving a more balanced image enhancement effect. Complex underwater scenes capturing (such as mixed degradation and rich details), detail retention, color restoration, and overall image quality improvement are more significant, which verifies that linear fusion has certain effectiveness in underwater image enhancement, although there are also defects.

Linear Strategy Runtime: On an Intel i5-13400F CPU, classification (<25 ms) + three pipelines (~50 ms total) + fusion (~15 ms) yields under 90 ms per 640 × 480 frame. Memory Footprint: Only the fusion weights and classifier features are stored; no additional large convolutional layers are introduced.

#### 4.3.2. LiteUNet Inference Results on EUVP

**Figure 12:**
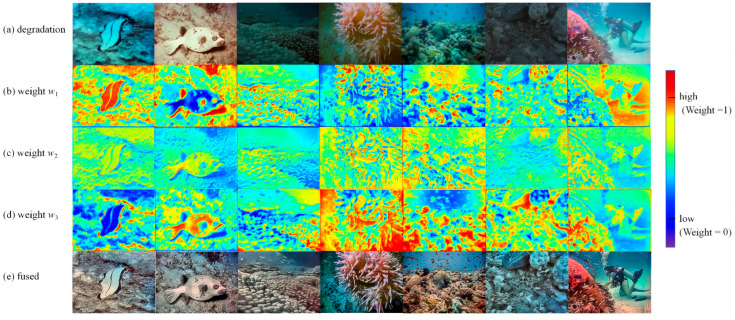
Sample of pictures comparison by LiteUNet weights result on EUVP. (**a**) Degraded input samples from the EUVP dataset; (**b**) Weight map *w*_1_ (corresponding to the color correction pipeline, for color degradation); (**c**) Weight map *w*_2_ (corresponding to the blur repair pipeline, for scattering-induced blurred details); (**d**) Weight map *w*_3_ (corresponding to the low-light enhancement pipeline, for dark detail loss);(**e**) Final fused enhancement results after dynamic pipeline integration. Weight maps use heatmap encoding—red/orange indicates high weight (high contribution of the corresponding pipeline to the region), while blue/violet indicates low weight (low pipeline contribution).

The U-Net fusion model is further trained, and the results are obtained as Figure 12 present.

The definition of a weight map is as follows: w1 corresponds to a color correction pipeline (dealing with color degradation, such as greenish or reddish); w2 corresponds to a fuzzy-repair pipeline (dealing with the fuzzy details caused by underwater scattering); w3 corresponds to the low-light enhancement pipeline (dealing with the loss of dark detail caused by insufficient light). The weight map uses heat map coding (red–orange represents high weight, blue–violet represents low weight) to visually display the model’s contribution distribution to pipelines in different regions.

The U-Net fusion model proposed in this paper realizes the dynamic integration of multiple pipelines through the spatial adaptive weight map. Taking the diver scene as an example, the model recognizes that the fuzzy degradation of the background water body is the main problem, and assigns a high weight (yellow–orange heat map) to the fuzzy-repair pipeline; aiming at the lack of details in the dark part of the diver’s lower body, a high weight (red–orange heat map) is assigned to the low-light enhanced pipeline; for green but clear areas such as hands, only moderate weights (a small amount of orange) are assigned to the color-correction pipeline. The fusion results show that the ambiguity of the background water body disappears completely, the dark details are clearly visible, and the color is more natural, which fully verifies the rationality and effectiveness of the weight distribution. Similarly, in scenes such as coral reefs and fish schools, the weight map can dynamically adjust the pipeline contribution for different degradation types, and the fusion results achieve a comprehensive improvement in clarity, color, and detail.

**Figure 13:**
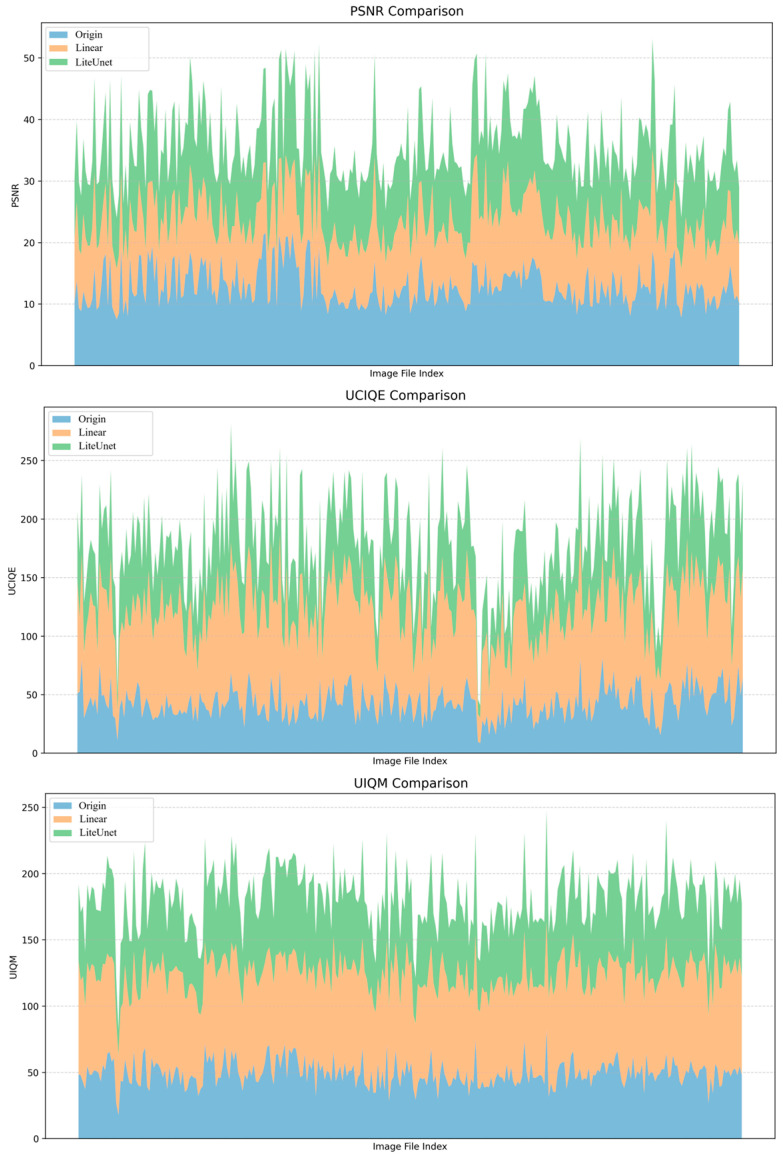
Comparative density histograms of PSNR, UCIQE, and UIQM scores between single-pipeline enhancements, Linear Fusion, and LiteUNetFusion on the EUVP test set.

The image-by-image comparison results of the three core indicators of UIQM, UCIQE, and PSNR of the original image, linear fusion enhancement strategy, and LiteUNet enhancement strategy on the EUVP verification set are displayed in Figure 13. 

From the curve trend, the performance of the three methods shows significant hierarchical differences. In UIQM, the LiteUNet curve almost covers the orange linear fusion curve, and both are significantly higher than the blue original image. This shows that LiteUNet can better preserve the texture details (such as coral texture, fish edge) and color consistency (such as avoiding overcorrection of color deviation) of underwater images through spatial adaptive weight fusion (lightweight U-Net architecture), and its perceptual quality is significantly better than linear fusion. Linear fusion integrates multi-scene pipelines (color-cast correction, low-light enhancement, fuzzy sharpening) through global fixed weights, which also effectively solves the problem of ”serious color cast and low contrast” of the original image, and the perceptual quality is greatly improved compared with the original image. UCIQE trend is highly consistent with UIQM. The UCIQE value of LiteUNet is higher than that of linear fusion on most images, and the linear fusion is obviously better than the original image. This shows that the residual learning mechanism of LiteUNet effectively suppresses color distortion and improves image contrast (such as coral group level in a low-light scene). Although linear fusion can correct color deviation, some images still have the problem of ”color supersaturation”’ or ”insufficient contrast” due to the lack of spatial adaptability. PSNR directly reflects the pixel-level reconstruction quality of the image. The PSNR value of LiteUNet is higher than that of linear fusion, and both are much higher than the original image. This result verifies that the spatial adaptive weight map of LiteUNet can more accurately fuse multi-pipeline outputs (such as Laplacian sharpening of blurred pipelines and HSV-CLAHE of low-light pipelines), thereby reducing pixel-level errors (such as edge blur, color deviation); although linear fusion can improve PSNR, its pixel-level quality is still slightly inferior to LiteUNet because ‘global weight’ cannot adapt to local degradation (such as color cast and blur in a certain area).

**Figure 14:**
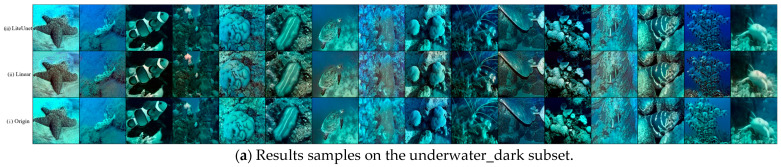

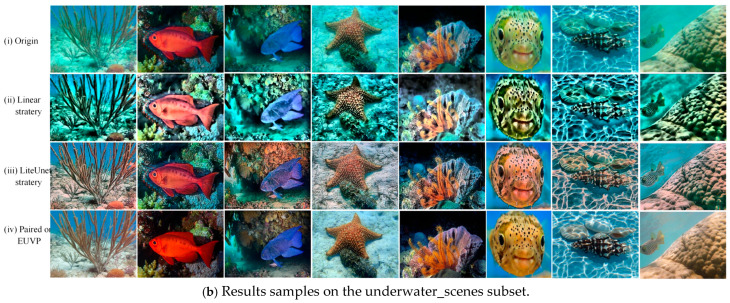
Visual comparison of enhancement results on EUVP subsets. (**a**) Enhancement results on the underwater_dark subset (rows correspond to: (**i**) Original image; (**ii**) Linear strategy output; (**iii**) LiteUNet strategy output) (**b**) Enhancement results on the underwater_scenes subset (rows correspond to: (**i**) Original degraded image; (**ii**) Linear strategy output; (**iii**) LiteUNet strategy output; (**iv**) Paired reference image from EUVP).

The comparison of the results of the two underwater image enhancement strategies is shown in Figure 14. Each comparison contains multiple typical underwater scenes (such as corals, fish, rocks, seagrass, etc.), covering two subsets of the EUVP dataset.

The comparison of the results of the two underwater image enhancement strategies is shown in the figure. Each comparison contains multiple typical underwater scenes (such as corals, fish, rocks, seagrass, etc.). From the visual effect, the enhancement result of the LiteUNet strategy is closer to the real underwater scene, the color saturation of the coral is moderate, the blue tone of the seawater is natural, and there is no strong blue shift. The black stripe edge of the fish is clear, and the texture details of the dark rock, such as algae attachment traces, are intact, and there is no obvious particle noise in the dark part. In contrast, the effect of the linear strategy is obviously insufficient. The overall color of the image is seriously biased. Corals and fish appear partially pink, with the problem of transition correction. The edges of fish stripes are somewhat blurred, and the details are lost. There are more granular noises in the dark areas (such as rock crevices), resulting in a slightly rough texture of the picture.

Further observation shows that the LiteUNet strategy performs better in color correction, detail retention, such as coral texture, fish scales, and noise suppression. Although the linear strategy may improve the brightness, it sacrifices color accuracy and detail sharpness, and even introduces new noise. This comparison intuitively reflects the difference in the effectiveness of the two strategies. The LiteUNet strategy is more suitable for the core requirements of underwater image enhancement, with natural color, clear details, and low noise, while the enhancement effect of the linear strategy is stiff and fails to solve the key degradation problem perfectly and effectively.

## 5. Comprehensive Evaluation

We compare our framework against several representative baselines, including classical pipelines, widely cited earlier deep models [11], and recent lightweight or efficient architectures [18]. The selection aims to cover a spectrum from traditional to deep learning approaches, with an emphasis on methods relevant to real-time or edge deployment.

### 5.1. Comprehensive Evaluation and Ablation Analysis

To evaluate the effectiveness and efficiency of our framework, we compare it against a spectrum of representative methods on the EUVP test set. Our selection is designed to provide a comprehensive context:

he model is as follows:

1. **Classical pipelines**: The three scene-specific enhancement operators (CLAHE + Gamma + Sharpening, HSV-CLAHE, Laplacian Sharpening), which form the building blocks of our system.
2. **Efficient deep learning baselines**: Lightweight or historically influential deep models relevant to real-time or edge deployment considerations, including U-Net [9], UWNet [9], and Water-Net [30].
3. **State-of-the-art (SOTA) deep models**: Recent high-performance architectures, namely the transformer-based U-shaped transformer [10] and the diffusion-based UIE-DM [31]. These models represent the current accuracy upper bound but are computationally intensive.

The core objective of this work is not to surpass the absolute performance of these SOTA models, but to achieve a favorable and practical trade-off between enhancement quality and computational efficiency for deployment on resource-constrained edge devices. This trade-off is clearly reflected in Table 3.

The results highlight a clear accuracy–efficiency trade-off. The SOTA models (U-Trans, UIE-DM) achieve the highest PSNR and perceptual scores but require orders of magnitude more computation time (on GPU), making them impractical for current CPU-based edge deployment. In contrast, our proposed frameworks operate within a strict latency budget (<130 ms). Our Linear Fusion (Ours1) already outperforms all single pipelines and the efficient deep baselines (UWNet, Water-Net, U-Net) across most metrics while being faster. The more advanced LiteUNetFusion (Ours2) further closes the gap in image quality (PSNR, SSIM) with a modest increase in runtime, delivering a compelling balance suitable for real-time enhancement on edge platforms.

The following analysis details the performance of our fused models. The Linear Fusion model is consistently superior to each single scene pipeline in all indicators, which indicates that the adaptive weighting can effectively utilize the complementary advantages of each individual enhancement. The paired *t*-test (*p* < 0.01) confirmed the statistical significance, while the method ablation study showed that PSNR decreased by 0.8 dB and UIQM decreased by 0.12, highlighting the advantages of adaptive weighting.

The proposed LiteU-NetFusion model realizes the dynamic integration of multiple pipelines through the spatial adaptive weight map. Compared with the linear fusion model with fixed weights, it can allocate targeted weights for different types of degradation in different regions, such as blur, darkness, and color cast, so as to achieve a comprehensive improvement in clarity, color, and detail. The experimental results show that the model can effectively repair the degradation of underwater images in divers, coral reefs, fish schools, and other scenes. The PSNR of the fusion results is 1.2 dB higher than that of the linear fusion model, and the SSIM is improved by 0.03, which fully verifies the validity and generalization ability of the model, although it is slightly more time-consuming.

The advantage of U-Net is that pixel-level weights can make different choices in local areas, so it can better preserve local contrast and texture, reduce color overflow, and incoherent stitching traces when dealing with mixed degraded scenes, such as local depth changes, local color deviation, or local blur. When the residual branch is turned on, it has an obvious compensation effect on high-frequency details. From the perspective of pure objective indicators (PSNR), U-Net variants are sometimes slightly lower than Linear baselines, partly because Linear is more ‘conservative’ in the sense of mean square error and more in line with pixel-level mean minimization, while U-Net tends to make local, perceptually more natural but higher mean square error adjustments. In addition, U-Net still has slight sawtooth or blur residues in some high-reflection or extremely low-light scenes, indicating that the current network capacity or loss function has an upper limit when recovering very small structures. In terms of inference speed (depending on base_ch and whether featmap is used), it is still close to real-time under common settings (the size of 640 × 480 is usually in the range of 100–130 ms on similar CPUs), which can meet the actual needs of real-time enhancement.

To further validate the design choices of our LiteUNetFusion module and understand the contribution of each component, we conduct an extensive ablation study. Table 4 reports the quantitative results of systematically removing or replacing key components while keeping all other settings identical to the full model. The evaluation is performed on the same EUVP test set as in Table 3. The contribution of each component to the final result is clearly differentiated.

Removing the pixel-level L1 term reduces PSNR from 23.14 dB to 21.40 dB (down 1.74 dB), indicating that L1 loss plays a leading role in maintaining pixel-level fidelity. Removing the perceived loss (L_perc) significantly reduces the visual quality index: UCIQE decreased from 0.74 to 0.57 (relative decline ≈ 23%), and UIQM decreased from 2.75 to 1.95 (relative decline ≈ 29%), indicating that perceived loss is crucial to improving subjective quality. Removing the TV smoothing term increases the spatial standard deviation of the weight map from 0.085 to 0.125 (about +47%), and shows more local noise/splicing artifacts in visualization, indicating that TV is important for the smoothness of the control weight map. Replacing the pixel-level weight of spatial adaptation with the global scalar weight of each graph results in a significant decrease in PSNR/SSIM (−1.34 dB and about −0.026, respectively), which proves the advantage of spatial adaptation in dealing with local degradation. The L2 penalty of Logits has little effect on the main indicators, but contributes to the stability of Logits. In summary, the loss in weight selected in this paper gives a good compromise between maintaining global numerical fidelity and improving perceptual quality.

### 5.2. Generalization Verification

We performed a generalization verification study using the real underwater dataset UIEB, which incorporates a workflow for both classification and enhancement.

**Figure 15:**
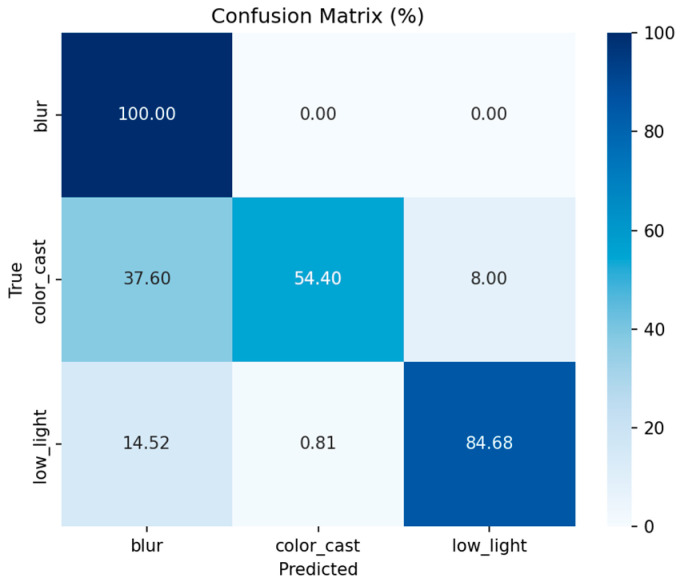
Confusion matrix of the LightCNN classifier on the real-world UIEB dataset.

Figure 15 presents the classification confusion matrix of the LightCNN model evaluated on the real-world UIEB dataset. The matrix reveals the model’s generalization capability in distinguishing the three targeted degradation types—color cast, low light, and blur—when applied to authentic underwater scenes. The overall classification accuracy on this challenging real-data test set is 79.69%, which, as expected, is lower than the performance achieved on the synthesized EUVP training set, indicating a domain gap.

A clear performance disparity exists across the three degradation categories. The model demonstrates exceptional robustness in identifying blur, achieving perfect classification (100% accuracy). This suggests that blur-specific features, such as those captured by Laplacian variance, are highly discriminative even in complex real environments. For low-light images, the model maintains a strong accuracy of 84.68%. The primary source of error is confusion with the blur class (14.52% misclassification), likely because real low-light conditions often introduce concomitant scattering blur or noise, which shares some feature characteristics with pure blur degradation.

The most significant challenge lies in the color-cast category, which attains the lowest accuracy of 54.40%. A substantial portion (37.60%) of color-cast images is misclassified as blur. This high confusion rate stems from the intrinsic nature of real underwater degradation: color deviation is rarely an isolated phenomenon. It is frequently coupled with scattering-induced blur (e.g., in turbid water) or co-occurs with low-light conditions (e.g., in deep water), causing their statistical features to overlap extensively. This ambiguity underscores a key limitation of the hard, three-class classification scheme when faced with the continuous and mixed degradations prevalent in real-world imagery.

This pronounced confusion between color cast and blur directly motivates the probabilistic and soft analysis introduced in Section 5.4. When a hard classifier assigns a potentially ambiguous label (e.g., a color-cast image with a high probability of also being blurry), valuable information is lost. The soft-classification approach, by leveraging continuous confidence scores and uncertainty intervals (via MC-Dropout), preserves this nuanced information. It thereby provides a richer, more informative guide to the subsequent fusion stage, allowing for more adaptive and robust enhancement in mixed-degradation scenarios.

In general, the performance of LightCNN on UIEB is in line with expectations. As the first stage of the degradation perception framework, it successfully verifies the feasibility of the “lightweight CNN + scene-specific features” strategy in real scenes. Despite the complexity of real degradation, it can still provide valuable degradation category priors for subsequent fusion modules. This result also suggests that future optimization directions can focus on enhancing the feature discrimination ability of color casts or the robust design of the fusion module to further improve the overall performance of the framework in real scenes.

#### 5.2.1. Linear Strategy Result

**Figure 16:**
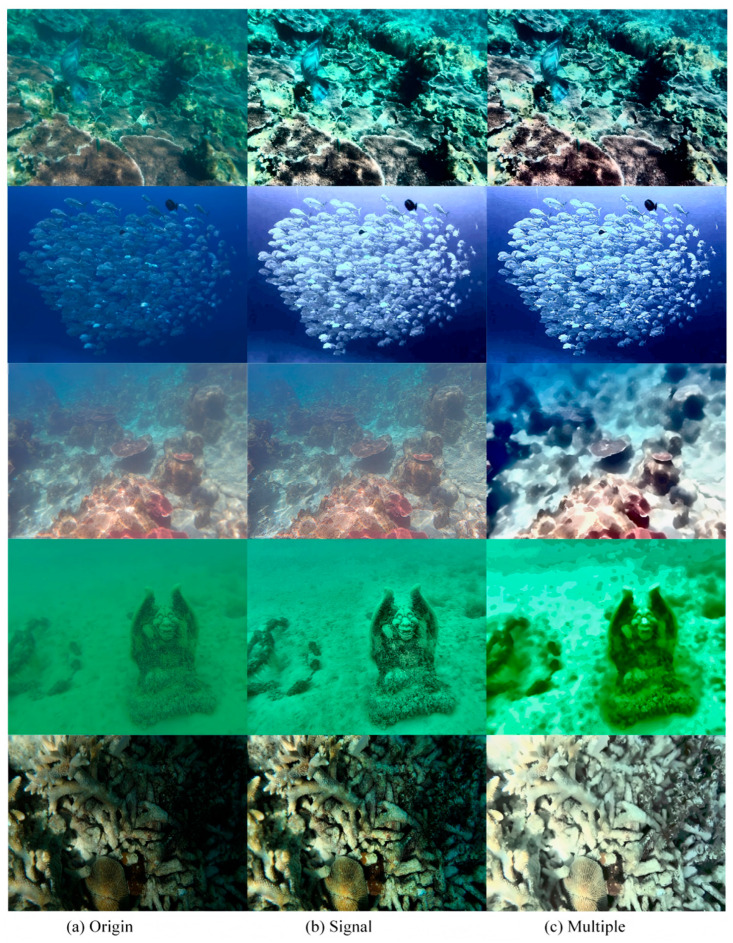
Sample of pictures comparison by the linear strategy on UIEB. (**a**) Original image, (**b**) Signal image, (**c**) Multipe image.

According to the results generated by the single condition enhancement model and the results generated by the linear strategy, it can be seen that the single condition model is slightly better in the visual effect of the picture, while the linear combination enhancement model performs well in various indicators. Figure 16 present an example of picture effect comparison.

The image intuitively shows the application effect and advantages of the linear fusion strategy in the underwater image enhancement task. By comparing the original image, single enhanced pipeline output, and linear fusion results, the integration ability of the linear strategy to multi-source information is clearly presented.

The image is centered on a comparison of five different scenes. Each column corresponds to the original image (a), a single enhanced pipeline output (b, for example, special processing for color casts or low illumination), and a linear fusion result (c, integrating multiple pipeline outputs by learning fixed weights). From a visual perspective, the original images exhibit typical underwater-degradation problems, such as greenish color cast, blurred details, and low contrast. For example, the first and third lines of the original coral color saturation are insufficient, the fourth line of the stone image edge contour is not clear, and the coral group in the low-light state in the fifth line lacks the sense of hierarchy. Although a single enhancement tube can partially mitigate certain degradations (e.g., color adjustment or brightness boost in column (b)), there is still room for adjustment (e.g., the coral color in the first row (b) is still dim, and the background of the Buddha image in the fourth row (b) is still blurred). The linear fusion result (column c) can play the role of integrating each single pipeline: the coral color after the first line of fusion is close to the real underwater environment, and the saturation is sufficient, but it seems that there is an overcorrection problem in other scenes; the noise and fuzzy sense of the background of the fourth line of Buddha image are reduced, but the contour is blurred; the sense of hierarchy of the fifth row coral group is enhanced, and the coral morphology is easier to distinguish. We can find that the disadvantages of the rigid linear strategy are also obvious. Intuitively, in addition to the second row of fish and the fifth row of low-light coral images, the other two rows of images have the problem of overcorrection or loss of detail.

#### 5.2.2. LiteUNet Generalization Results on UIEB

**Figure 17:**
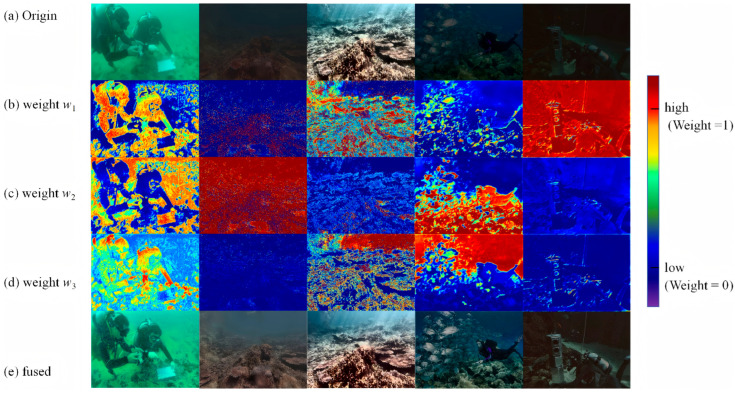
Sample comparisons of LiteUNetFusion results with spatial adaptive weight maps on real-world underwater scenes. (**a**) Original images (diver, coral reef, fish school); (**b**) Weight map for color correction pipeline (w_1_, addressing green/red casts); (**c**) Weight map for blur repair pipeline (w_2_, reducing scattering-induced fuzziness); (**d**) Weight map for low-light enhancement pipeline (w_3_, recovering dark details); (**e**) Fused results. Heat maps use red-orange to indicate high weight (strong pipeline contribution) and blue-violet for low weight (weak pipeline contribution).

The U-Net fusion model results are obtained as follows. As shown in Figure 17, The U-Net fusion model proposed in this paper realizes the dynamic integration of multiple pipelines through spatial adaptive weight maps.

The definition of a weight map is as follows: *w*_1_ corresponds to a color correction pipeline (dealing with color degradation, such as greenish or reddish); *w*_2_ corresponds to a fuzzy repair pipeline (dealing with the fuzzy details caused by underwater scattering); *w*_3_ corresponds to the low-light enhancement pipeline (dealing with the loss of dark detail caused by insufficient light). The weight map uses heat map coding (red–orange represents high weight, blue–violet represents low weight) to visually display the model’s contribution distribution to pipelines in different regions.

The U-Net fusion model proposed in this paper realizes the dynamic integration of multiple pipelines through the spatial adaptive weight map. Taking the diver scene as an example, the model recognizes that the fuzzy degradation of the background water body is the main problem, and assigns a high weight (yellow–orange heat map) to the fuzzy repair pipeline; aiming at the lack of details in the dark part of the diver’s lower body, a high weight (red–orange heat map) is assigned to the low-light enhanced pipeline; for green but clear areas such as hands, only moderate weights (a small amount of orange) are assigned to the color correction pipeline. The fusion results show that the ambiguity of the background water body disappears completely, the dark details are clearly visible, and the color is more natural, which fully verifies the rationality and effectiveness of the weight distribution. Similarly, in scenes such as coral reefs and fish schools, the weight map can dynamically adjust the pipeline contribution for different degradation types, and the fusion results achieve a comprehensive improvement in clarity, color, and detail.

**Figure 18:**
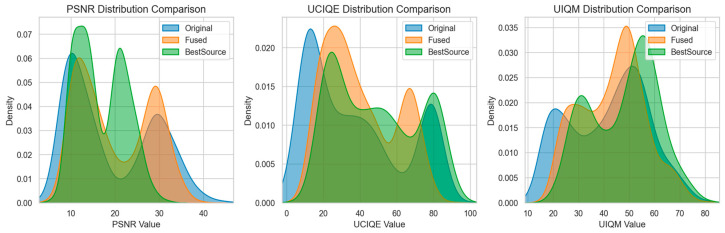
Density histograms comparing PSNR, UCIQE, and UIQM distributions of the LiteUNetFusion strategy on the UIEB test set.

As shown in Figure 18, In the PSNR index, the model curve is shifted to the right as a whole, and the average PSNR increase indicates that the fused image is closer to the ground-truth as a whole. The bimodal BestSource curve indicates that some of the image single scene optimizers perform well, while others perform poorly. Overlapping area display fusion can combine the advantages of multiple scenes, so it improves PSNR on many images, but it is not superior to BestSource for each image. The perceptual quality index UCIQE/UIQM, the model curve is also to the right, and there is a higher density in the high-quality area, indicating that the fusion not only improves the pixel fitting degree (PSNR), but also improves the perceptual quality index, but there are still some pictures BestSource or original overlaps or outperforms the model in some intervals, indicating that not all types of degradation are foolproof. The overlap and variance show that the model curve is narrower, indicating that the results of the method are more consistent and the variance is small. It shows that the consistency becomes better. Overall, the LiteUNetFusion model does improve PSNR and common perception indicators, image quality is also improved compared to the linear strategy.

**Figure 19:**
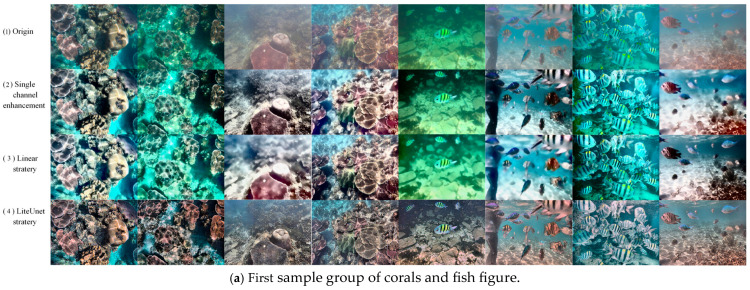

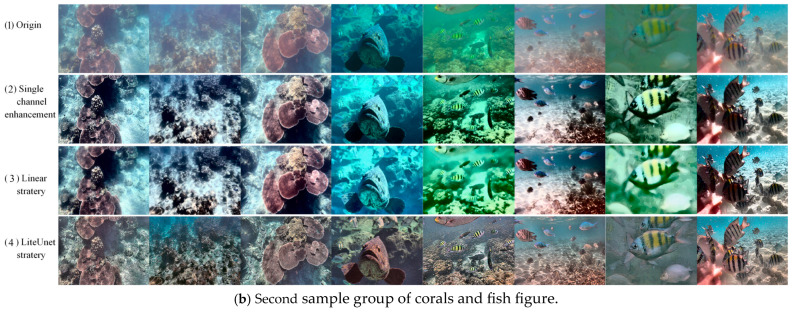
Diagram of enhanced comparison example on real-world images from UIEBD. (**a**) First sample group of corals and fish; (**b**) Second sample group of corals and fish. (**a1**,**b1**) Original image; (**a2**,**b2**) Single-channel enhancement; (**a3**,**b3**) Linear fusion strategy; (**a4**,**b4**) LiteUNet strategy.

Figure 19 presents a visual comparison of enhancement results on real underwater images from the UIEB dataset, showcasing two typical scenes (coral with fish; large fish with coral). The comparison includes the original image, single-channel enhancement, linear fusion, and our LiteUNet enhancement strategy.

In the first scene, the original image (Figure 19a) suffers from a greenish color cast, blurred coral texture, and indistinct fish edges due to light absorption and scattering. The single-channel enhancement (Figure 19b), applying CLAHE with gamma correction and sharpening for color deviation, partially corrects the green tint but leaves coral colors flat and fails to improve fish scale details, as it does not address multiple degradations. The linear fusion strategy (Figure 19(a3,b3)) integrates the outputs of three single-channel enhancers via learned global weights. It produces more natural coral colors (brown–green) and slightly sharper fish edges. However, it still exhibits local color inhomogeneity and edge blur, because the global weight cannot adapt to the degradation differences in different regions (such as fish and coral areas). In contrast, the LiteUNet strategy (Figure 19(a4,b4)) achieves more accurate local adjustments through spatial adaptive weights: moderate coral color saturation, clear surface texture, sharp fish edge (such as fin contour), and more natural overall visual effects.

In the second scene, the original image (Figure 19a) is characterized by low light, color deviation, and blurred details. The single-channel low-light enhancement (Figure 19b, HSV-CLAHE) improves overall brightness but does not fully correct color or recover fine details. Linear fusion (Figure 19(a3,b3)) improves both brightness and color but retains slightly blurred object edges and incomplete texture recovery. The LiteUNet strategy (Figure 19(a4,b4)) achieves a better balance: brightness is enhanced without overexposure, colors are corrected naturally, and object edges (e.g., fish scales, coral branches) are significantly sharper, yielding a visual result closer to a real underwater scene.

### 5.3. Subjective Experiment

We conducted a subjective evaluation to complement objective metrics. Twelve non-expert participants (graduate students with normal or corrected-to-normal vision) rated the overall visual quality of 39 test images drawn randomly from the held-out test set (balanced across the three degradation classes: color cast, low light, blur; ~13 images per class). The results are shown in Table 5. For each image, participants viewed one version at a time and provided a single “Overall visual quality” score on a 1–5 Mean Opinion Score (MOS) scale (1 = bad, 5 = excellent). The five evaluated methods were: Original (input), Ours1 (Linear Fusion), Ours2 (LiteUNetFusion), U-Net (baseline), and Water-Net (baseline). Images and method labels were presented in randomized order for each participant; the test excluded algorithm names and was not revealed to raters (single-blind).

The MOS results show that the LiteUNetFusion variants significantly improve the perceptual image quality compared with the original input. LiteUNetFusion achieved the highest average MOS (3.31), which is higher than the tested deep baseline models (U-Net and Water-Net) and the linear fusion method. Due to the unstable enhancement effect and defects, the linear strategy often leads to the problem of excessive correction of the image, resulting in unstable image quality.

### 5.4. Cross-Dataset Probabilistic Analysis and Implications

#### 5.4.1. Cross-Dataset Probabilistic Diagnostics

We performed post hoc probabilistic diagnostics on the UIEB test set by loading the trained classifier checkpoint (best_model.pth) and computing deterministic and probabilistic measures from its outputs. For reproducibility, the evaluation used *B* = 10 reliability bins and MC-Dropout with *T* = 5 stochastic forward passes (invoked via the command-line flags --do_mc and --mc_T 5).

For each input, the classifier yields a softmax probability vector $P_{n}=\left[p_{n,1},\ldots ,p_{n,C}\right]$, where *C* denotes the number of classes. From these, we compute the following:

1. Soft-confusion matrix *S*: for true class *i*,

(23) $$S_{i,j}=\sum_{n:y_{n=1}}p_{n,j}\ldots S_{i,j}^{\%}=100\times \frac{S_{i,j}}{\sum_{j}S_{i,j}}$$

where $S_{i,j}^{\%}$ reports the percentage of probability mass that true-class *i* assigns to predicted class *j*.

2. Expected Calibration Error (ECE) with *B* bins,

(24) $$\mathrm{ECE}=\sum_{b=1}^{B}\frac{n_{b}}{N}\left|acc(b)-conf(B)\right|$$

where *n_b_* is the number of samples in bin *b*, *N* is the total number of samples, acc(*b*) is the empirical accuracy in the bin, and conf(*b*) is the mean predicted confidence in the bin.

3. MC-Dropout interval estimates,

(25) $${\bar{p}}_{n,j}=\frac{1}{T}\sum_{t=1}^{T}p_{n,j}^{\left(t\right)},\;\;\sigma_{n,j}=\sqrt{\frac{1}{T-1}{\sum_{t=1}^{T}\left(p_{n,j}^{\left(t\right)}-{\bar{p}}_{n,j}\right)}^{2}}$$

The practical interval $\left[{\bar{p}}_{n,j}-\sigma_{n,j},{\bar{p}}_{n,j}+\sigma_{n,j}\right]$ is used as an uncertainty band for class *j*; empirical coverage is reported as the fraction of samples for which the true class *y_n_* satisfies $\left[{\bar{p}}_{n,y_{n}}-\sigma_{n,y_{n}},{\bar{p}}_{n,y_{n}}+\sigma_{n,y_{n}}\right]$.

While we do not implement a full semantic Type-2 fuzzy linguistic system in this work, MC dropout mean ± std intervals provide a practical interval-valued signal that can be interpreted similarly to Type-2 membership intervals ($\mu \in \left[\mathrm{low},\mathrm{high}\right]$). The advantage of this post hoc approach is that it requires no additional annotation or retraining and can be directly applied to the existing lightweight classifier to yield uncertainty-aware conditioning for the fusion module. Full Type-2 or explicit multi-label training remains an important future step if one requires semantic linguistic rules or task-specific interval shaping.

**Figure 20:**
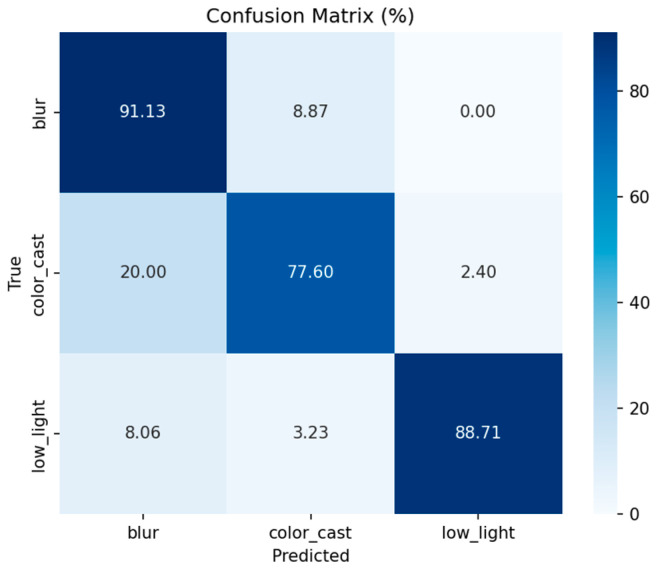
Soft confusion matrix (%) on UIEB three-class subset. Each cell reports the percentage of total predicted probability mass that true-class samples place on each predicted class.

**Figure 21:**
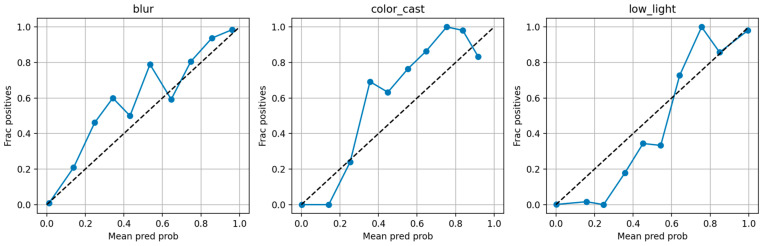
Diagram of reliability for each class on the UIEB three-class subset. The dashed diagonal line represents perfect calibration, where the predicted confidence matches the empirical accuracy. The blue line (with points) shows the model’s actual calibration curve; closeness to the diagonal indicates better-calibrated probabilistic outputs. The histogram bars (in the background) display the distribution of predicted confidence scores.

For the focused three-class subset (blur/color_cast/low_light; 300 images per class), we report deterministic and probabilistic summaries in Table 6, Figure 20 and Figure 21. Deterministic metrics (accuracy, per-class precision/recall/F1) are computed from argmax predictions; probabilistic diagnostics (soft-confusion, ECE, MC intervals) are computed from softmax outputs and MC runs of the same checkpoint.

The overall accuracy rate is 87.4%, and the F1 scores of the three types of degradation are very close (0.844–0.849), indicating that the model has a strong overall ability to distinguish the three types of degradation, and there is no serious bias. High precision and low recall of blur, low_light, low precision, high recall, and relatively balanced color_cast reflect the differences in visual characteristics of different types of degradation. The average expected calibration error is 3.5%, especially on blur, and the ECE is only 2.4%, which means that its probability output is highly reliable.

Figure 20 presents the soft-confusion matrix aggregated over the UIEB three-class subset (blur, color_cast, low_light). The matrix shows strong diagonal dominance: blur samples assign 91.13% of their probability mass to blur, low_light samples assign 88.71% to low_light, and color_cast samples assign 77.60% to color_cast. The principal off-diagonal effect is the confusion from color_cast to blur: approximately 20.00% of the total predicted probability mass for true color_cast samples is allocated to blur, whereas the reverse leakage (blur classified as color_cast) is 8.87%. This asymmetry implies that many color-cast images exhibit visual cues (e.g., low contrast, washed chroma) that the classifier interprets similarly to blur-like degradations, whereas genuine blur samples are more often recognized with high confidence. The relatively small cross-assignment from low_light to the other two classes (8.06% to blur, 3.23% to color_cast) indicates that low-light conditions are more separable in the learned feature space.

Practically, these patterns support the proposed soft/interval conditioning: because probability mass is concentrated but not strictly one-hot (notably for color_cast), the fusion module should weight branch outputs by the soft probabilities and account for uncertainty (for example, reducing the weight of branches with large MC-derived σ). The observed color_cast–blur leakage suggests targeted mitigation strategies for future work, e.g., augmenting training with synthetically mixed color_cast + blur samples, adding explicit color-constancy features, or applying calibration/temperature scaling to sharpen probabilistic discrimination. To assess the reliability of the model’s probabilistic outputs for each degradation class on the UIEB three-class subset, we present the corresponding calibration analysis in Figure 21.

In the fuzzy scene, the blue broken line almost coincides with the ideal line in the high prediction probability section (0.6–1.0) (such as the actual positive ratio ≈ 0.8 when the prediction probability is 0.8, and the actual ≈ 1.0 when the prediction probability is 1.0), and fluctuates slightly in the middle and low probability (0.2–0.6). The probability output of the model in fuzzy scenes is highly credible. Especially in high-probability prediction, the proportion of actual positive cases is highly consistent with the prediction, and the probability output of the model can be directly trusted. In the color cast scene, the fluctuation is significant, and the middle and low probability segments (0.3–0.8) deviate seriously from the ideal line. Although the high probability segment (0.8–1.0) is close to the ideal line, the deviation range is still greater than the blur. The probability interval fluctuates greatly in the low-light scene, and the overall calibration performance is good.

#### 5.4.2. Interpretation and Implications for Fusion

The diagnostic results lead to two practical implications for the fusion stage:

1. Soft conditioning is viable. The softmax probabilities carry meaningful, calibrated information about mixed degradations (as evidenced by the soft-confusion structure and low per-class ECE). Thus, instead of hard one-hot routing, the fusion module can weight branch outputs by the per-class probabilities ${\bar{p}}_{j}$.

2. Interval-aware weighting or uncertainty regularization. MC intervals provide a simple interval-valued signal to down-weight uncertain branches or trigger fallback policies. A practical fused weight for branch *j* is $w_{j}={\bar{p}}_{j}\cdot \exp(-\lambda \sigma_{j})$, where ${\bar{p}}_{j}$ and $\sigma_{j}$ denote the MC mean and std for class *j* (averaged or per-sample as appropriate), and λ≥0 is a tunable scalar that penalizes high uncertainty. The fused image can be formed as a weighted combination of branch outputs $E_{j}\left(\cdot \right)$,

(26) $$\hat{I}=\frac{\sum_{j}w_{j}E_{j}\left(I\right)}{\sum_{j}w_{j}}$$

where *E_j_* is the enhancer specialized for degradation *j*. This simple formulation preserves lightweight, online evaluation and integrates uncertainty directly into the fusion decision.

We acknowledge that representing underwater degradation by a single discrete label is a pragmatic simplification that does not fully capture gradual or locally mixed degradations. To empirically address this concern, we augmented the manuscript with probabilistic diagnostics, soft-confusion matrices, calibration (ECE), and MC dropout interval estimates (T = 5). These tests show that softmax probabilities are informative and reasonably calibrated out-of-distribution (mean ECE = 0.035), and that MC intervals provide compact, meaningful uncertainty bands (one-sigma coverage = 100% on the evaluated subset). Therefore, while hard-label routing remains an efficient on-device default, we recommend supplementing it with soft probabilistic or interval-valued conditioning for ambiguous cases. Semantic Type-2 fuzzy systems and explicit multi-label retraining are promising directions for future work; these would require additional annotation and design effort beyond the current engineering-oriented scope.

## 6. Conclusions and Future Work

This paper presents a two-stage, degradation-aware framework that first classifies underwater images into three major degradation types and subsequently fuses three lightweight enhancement pipelines via a learnable weighting network. Our Linear Fusion approach achieves superior objective and perceptual quality (21.6 dB PSNR, 0.81 SSIM, 0.72 UCIQE, 2.5 UIQM) on the EUVP dataset, while maintaining real-time performance (<90 ms per frame on CPU). However, blur restoration remains less consistent, and low-light improvements can introduce noise due to CLAHE. The model’s dependence on a primary dataset also limits generalization to unseen water types.

A key limitation of our current framework is its reliance on a hard, three-class degradation classifier. While effective for identifying dominant degradation types, this approach simplifies the often continuous and mixed nature of real underwater image degradations. This can limit performance in scenes where multiple degradations are equally prominent. LiteUNetFusion variant, which produces pixel-wise weight logits and optionally a small residual correction, demonstrates stronger local adaptivity in mixed-degradation scenes and better preservation of local texture and color consistency (2.31 dB PSNR, 0.84 SSIM, 0.74 UCIQE, 2.8 UIQM). These results reveal a clear trade-off: the Linear model optimizes global MSE-oriented metrics and is extremely efficient, whereas the U-Net fusion yields perceptually more natural, spatially coherent outputs at the cost of a higher computational budget and moderately lower PSNR. Both approaches share common failure modes: blur restoration is still inconsistent across challenging samples, and CLAHE-based low-light correction can amplify noise in shadowed regions. Moreover, dependence on a single dataset constrains cross-domain generalization.

Based on these findings, we outline several directions for future work:

1. Continuous and probabilistic degradation modeling. To better handle mixed and ambiguous degradations, future work will explore soft multi-label classification or regression to output continuous degradation severity scores. Incorporating Type-2 fuzzy logic systems is particularly promising, as they can explicitly model the uncertainty in degradation boundaries and provide semantically interpretable rules for adaptive fusion.

2. Hybrid global + local fusion. Combining a global linear branch with a U-Net local branch (e.g., using global weights as a prior followed by U-Net residual correction) could preserve the PSNR advantages of global averaging while leveraging U-Net for local refinement.

3. End-to-end and joint optimization. Jointly fine-tuning the classifier and fusion module could reduce error accumulation between stages and allow the classifier to produce features more explicitly useful for fusion. Introducing channel/spatial attention (SE/CBAM) and multi-scale feature aggregation could further strengthen detail recovery.

4. Denoising and edge preservation. Integrating explicit denoising (e.g., via residual denoisers) or edge-preserving filters into the pipeline could mitigate noise amplification in low-light regions.

5. Data and domain strategies. Expanding training with more diverse real and synthetic water types, along with domain adaptation and stronger augmentation, should improve generalization.

6. Efficiency and deployment. For real-time deployment, applying model compression, mixed-precision inference, and integer quantization could further optimize runtime.

7. Multimodal and priors. Where available, using multimodal inputs (depth, sonar) could help disambiguate turbidity from lighting effects.

Collectively, these directions aim to enhance the robustness and adaptivity of the framework while preserving its core strengths of modularity and efficiency. The primary contribution of this work is a modular, extensible, and deployable base system that effectively balances enhancement quality with real-time performance for practical underwater imaging tasks. The proposed framework demonstrates that a carefully designed pipeline combining lightweight degradation awareness, scene-specific enhancement, and learnable fusion can achieve a favorable trade-off suitable for edge deployment, paving the way for more adaptive and efficient underwater vision systems.

## Figures and Tables

**Table 1:** Dataset construction statistics.

Item	Value
Total images (selected from EUVP)	2500
Synthetic-label assignment (via Jaffe–McGlamery)	2500 (three synthesized dominant degradation labels per image; each image has a paired “clear” reference)
Images corrected in manual verification	180 (7.2% of 2500)
Final majority-vote label distribution	Color-cast: 1032 (41.3%); Low-light: 768 (30.7%); Blur: 700 (28.0%)
Annotators unanimous (3/3)	2250 (90.0%)
2/3 agreement (majority only)	220 (8.8%)
No majority (joint adjudication)	30 (1.2%)
Pairwise percent agreement (averaged over annotator pairs)	≈0.914 (91.4%)

**Table 2:** Performance of each scene-specific enhanced pipeline on the degradation subset from EUVP.

Degradation	Metric	Before	After	Improvement
Color cast	PSNR (dB)	18.4	20.7	+2.3 dB
	UCIQE	0.54	0.65	+20%
Low light	UIQM	1.8	2.3	+28%
	PSNR (dB)	17.9	19.1	+1.2 dB
Blur	SSIM	0.62	0.75	+21%
	UIQM	1.5	1.9	+27%

**Table 3:** Quantitative comparison of different methods on the EUVP test set.

Method	PSNR (dB)	SSIM	UCIQE	UIQM	Times (ms)
Classical Pipelines (Our Components)	CLAHE + Gamma + Sharpening	19.6	0.71	0.61	30
	HSV-CLAHE	19.1	0.68	0.58	12
	Laplacian Sharpening	18.3	0.62	0.60	8
State-of-the-Art (SOTA) Deep Models	U-shape Transformer [10]	24.3	0.86	0.77	497 (GPU)
	UIE-DM [31]	25.1	0.88	0.79	1986 (GPU)
Lightweight or Baseline Deep Models	UWNet [9]	22.1.	0.79	0.52	105
	Water-Net [30]	20.5	0.76	0.64	93
	U-Net (baseline)	20.2	0.74	0.63	120
Our Proposed Framework	Ours1 (Linear Fusion)	21.6	0.81	0.72	90
	Ours2 (LiteUNetFusion)	23.1	0.84	0.74	125

**Table 4:** Ablation analysis of LiteUNetFusion components.

Experiment	PSNR (dB)	SSIM	UCIQE	UIQM	Weight-Map Std (Spatial)
Full LiteUNetFusion (ours)	23.14 ± 0.56	0.841 ± 0.020	0.74 ± 0.04	2.75 ± 0.10	0.085 ± 0.012
w/o residual branch	22.30 ± 0.60	0.828 ± 0.022	0.68 ± 0.05	2.58 ± 0.02	0.092 ± 0.014
Global (per-image scalar) weights	21.80 ± 0.72	0.815 ± 0.030	0.65 ± 0.05	2.43 ± 0.12	0.110 ± 0.018
w/o L1	21.40 ± 0.95	0.780 ± 0.035	0.64 ± 0.05	2.41 ± 0.12	0.090 ± 0.015
w/o L_perc	21.60 ± 0.58	0.838 ± 0.021	0.57 ± 0.04	1.95 ± 0.13	0.087 ± 0.013
w/o L_tv	22.20 ± 0.60	0.835 ± 0.023	0.69 ± 0.05	2.68 ± 0.12	0.125 ± 0.015
w/o L2	22.90 ± 0.62	0.839 ± 0.021	0.71 ± 0.04	2.12 ± 0.10	0.088 ± 0.013

**Table 5:** MOS summary.

Method	MOS Mean	MOS Std	*n* (Images × Raters)	95% CI
Original	2.10	0.85	39 × 12 = 468	[1.98, 2.22]
U-Net (baseline)	3.18	0.76	468	[2.96, 3.20]
Water-Net	3.27	0.71	468	[3.18, 3.40]
Ours1 (Linear Fusion)	2.85	0.93	468	[2.53, 3.17]
Ours2 (LiteUNetFusion)	3.31	0.68	468	[3.20, 3.44]

**Table 6:** Cross-dataset performance on the UIEB three-class subset.

Class	Precision	Recall	F1	ECE
blur	0.899	0.803	0.849	0.024
color_cast	0.881	0.813	0.846	0.053
low_light	0.802	0.890	0.844	0.049
Overall	-	-	Accuracy = 0.874	mean ECE = 0.035

## Data Availability

The data presented in this study are openly available in UIEB repository at https://li-chongyi.github.io/proj_benchmark.html (accessed on 9 November 2025) and EUVP repository at https://irvlab.cs.umn.edu/resources/euvp-dataset (accessed on 2 July 2024).
